# Selection strengthens the relationship between plant diversity and the metabolic profile of *Plantago lanceolata*

**DOI:** 10.1111/nph.70340

**Published:** 2025-07-24

**Authors:** Pamela Medina‐van Berkum, Francesca De Giorgi, Beate Rothe, Walter Durka, Jonathan Gershenzon, Christiane Roscher, Sybille B. Unsicker

**Affiliations:** ^1^ Department of Biochemistry Max Planck Institute for Chemical Ecology Jena 07745 Germany; ^2^ Department of Physiological Diversity Helmholtz Centre for Environmental Research – UFZ Leipzig 04318 Germany; ^3^ German Centre for Integrative Biodiversity Research (iDiv) Halle‐Jena‐Leipzig Leipzig 04103 Germany; ^4^ Department of Community Ecology Helmholtz Centre for Environmental Research – UFZ Halle 04318 Germany; ^5^ Plant‐Environment‐Interactions Group, Botanical Institute Kiel University Kiel 24118 Germany

**Keywords:** biodiversity, chemodiversity, eco‐metabolomics, experimental grasslands, Jena Experiment, phytometer, *Plantago lanceolata*

## Abstract

- Plant species diversity enhances community productivity, but how plant diversity impacts the metabolome of individual plants and the underlying eco‐evolutionary processes remains unclear. This study investigated how plant species diversity and selection for growing in different diversity environments affect the leaf metabolome of *Plantago lanceolata.*
- We compared the metabolites of plants derived from those that had been *selected* in the ‘Jena Experiment’ for 17 yr in plant communities with differing plant diversity with the metabolites of *naïve* plants not subjected to this selection. The metabolic profiles of *selected P. lanceolata* plants were also compared after growing in experimental environments varying in plant diversity, soil history and community plant history.
- Volatile compound diversity in *P. lanceolata* decreased with plant species richness (SR), primarily due to phenotypic plasticity rather than selection. Soil history further strengthened this relationship. Conversely, non‐volatile compound diversity increased with plant SR, but only in phytometers subjected to diversity‐driven selection. These effects were more pronounced when plants shared soil–plant history with their community.
- In summary, our study revealed that both plastic and adaptive responses shape the metabolome of *P. lanceolata* in relation to plant diversity, with these effects becoming stronger as plant and soil communities mature.

## Introduction

The current concerns about the loss of global biodiversity have intensified efforts to understand the mechanisms mediating the relationships between biodiversity and ecosystem functioning. Experimental studies on grassland biodiversity revealed that high plant diversity promotes plant community productivity and stability (Cardinale *et al*., 2007; Allan *et al*., 2013; Wagg *et al*., 2022). These effects strengthen over time as complementary interactions between species become more important (Cardinale *et al*., 2007), leading to more pronounced relationships between biodiversity and ecosystem functioning (Reich *et al*., 2012). Although several studies on species‐level responses to increased plant community diversity exist, they mostly focused on plant biomass production or plant morphological traits (Tilman *et al*., 1996; Lipowsky *et al*., 2011; van Moorsel *et al*., 2018). Only a few have investigated other important plant traits, such as specialized plant metabolites (e.g. Scherling *et al*., 2010; Mraja *et al*., 2011; Zuppinger‐Dingley *et al*., 2015; Ristok *et al*., 2023). This is of particular importance as plant phenotypes are shaped by the synthesis and subsequent accumulation of specialized metabolites in their organs during different developmental stages and in response to environmental cues.

Plant specialized (secondary) metabolites play essential roles in species interactions within communities as deterrents, toxins, attractants, or signals for other organisms and in resistance to abiotic stresses (Erb & Kliebenstein, 2020). By mediating biotic interactions, they can impact plant performance and survival. Specialized metabolites thus either act directly (as toxins and deterrents) or indirectly by attracting natural enemies of herbivores, often through the release of volatile organic compounds (VOCs). These metabolites can be constitutively present, providing a baseline level of defense (Wittstock & Gershenzon, 2002), or can be induced in response to biotic interactions or environmental factors, through increased production or *de novo* synthesis (Karban & Baldwin, 1997). This functional flexibility highlights the importance of plant chemodiversity, encompassing both the richness and composition of these chemicals, which can vary not only due to genetic differences but also in response to environmental pressures, such as herbivory and pathogen attacks or resource availability (Endara *et al*., 2023).

The environmental pressures a plant is exposed to also depend on plant community diversity. Several studies have shown that the increase in plant species richness (SR) leads to reduced pressure by antagonists such as root‐feeding nematodes, soil pathogens, and herbivorous arthropods (Wang *et al*., 2019; Barnes *et al*., 2020; Dietrich *et al*., 2021) while at the same time, mutualist interactions with mycorrhizal fungi, pollinators, and predators increase (Ebeling *et al*., 2011; Rottstock *et al*., 2014; Barnes *et al*., 2020; Dietrich *et al*., 2020). The varying selection pressures along plant diversity gradients are known to drive changes in plant phenotypes and genotypes over time (Zuppinger‐Dingley *et al*., 2014; Miehe‐Steier *et al*., 2015; De Giorgi *et al*., 2025), and it is expected that changes in antagonist and mutualist communities below‐ and aboveground across these gradients might influence patterns of plant resource allocation into defense.

Plants in high‐diversity communities may reduce their investment into defense due to increased growth (growth‐defense trade‐off hypothesis; Endara & Coley, 2011; Monson *et al*., 2022) and reduced attack by herbivores and pathogens (resource dilution hypothesis; Otway *et al*., 2005), resulting in reduced metabolome diversity. Furthermore, plant metabolic profiles might also be affected by associational effects driven by the identity of the surrounding plant community (Agrawal *et al*., 2006; Underwood *et al*., 2014). Consequently, metabolome responses may be compound‐specific rather than uniform, with variation in chemodiversity within the same plant species arising from phenotypic plasticity or genotype selection in response to the surrounding environment (Zuppinger‐Dingley *et al*., 2015). Phenotypic plasticity can take place within an organism's lifespan in response to its environment, while evolutionary adaptations occur over a time span of a few (Rauschkolb *et al*., 2022) to many generations (Nicotra *et al*., 2010). Previous research has reported both plastic and adaptive responses at the chemical level. For instance, Zuppinger‐Dingley *et al*. (2015) found that the different selection pressures in low‐ or high‐diversity communities led to adaptation in plant chemical traits of several grassland species over several generations. Furthermore, Miehe‐Steier *et al*. (2015) demonstrated that the concentration of the main defense compounds (iridoid glycosides) of *Plantago lanceolata* L. (ribwort plantain) shows a plastic response to plant SR with decreasing concentrations of aucubin and catalpol with increasing plant SR in their surroundings.

Another potential selective driver in plant communities of different diversity is the interaction between plant species and biotic and abiotic soil conditions, known as plant–soil feedback, in which soil communities modify the biotic and abiotic environment of plants, while plants create belowground legacies by altering the soil's biotic and abiotic properties (van der Putten *et al*., 2013). Over time, low‐diversity communities show an accumulation of specific plant antagonists and an imbalanced use of resources (Mommer *et al*., 2018; van Ruijven *et al*., 2020). By contrast, high‐diversity communities accumulate plant growth‐promoting organisms, as well as microbes that help to mobilize plant nutrients (Dietrich *et al*., 2021). As plant communities age, biodiversity effects on ecosystem functioning intensify (Amyntas *et al*., 2025). Soil history becomes an important agent, influencing selection processes in plants (Dietrich *et al*., 2021; De Giorgi *et al*., 2025). These processes can influence plant defense by modulating plant chemodiversity (Huberty *et al*., 2020; Ristok *et al*., 2023). It is important to note that such effects may depend on the type of compound involved. For instance, mycorrhiza formation in *P. lanceolata* reduced the emission of sesquiterpenes without affecting the concentration of iridoid glycosides in the leaves (Fontana *et al*., 2009). Similarly, soil legacy effects driven by plant SR influenced plant metabolites by altering soil biota (Ristok *et al*., 2019).

Plant metabolites play a crucial role in the establishment, development, and survival of plants (Erb & Kliebenstein, 2020). However, our understanding of how different environments shape heritable and adaptive metabolic profiles remains limited, particularly regarding the contrasting selective pressures acting on volatile and non‐volatile compounds. Long‐term biodiversity studies allow us to examine whether the influence of plant species diversity on the plant metabolome is due to the adaptation of plant species to different environments of origin or to phenotypic plasticity in response to the current environment. Here, we used *P. lanceolata* L., a short‐lived perennial, as a model species in a ‘phytometer’ experiment with planted individuals within a long‐term grassland biodiversity experiment (The Jena Experiment; Roscher *et al*., 2004). This study aimed to disentangle the roles of adaptation and plasticity in shaping the metabolic responses of *P. lanceolata* to plant community diversity, with a focus on how these processes contribute to both volatile and non‐volatile compound diversity.

**Fig. 1:**
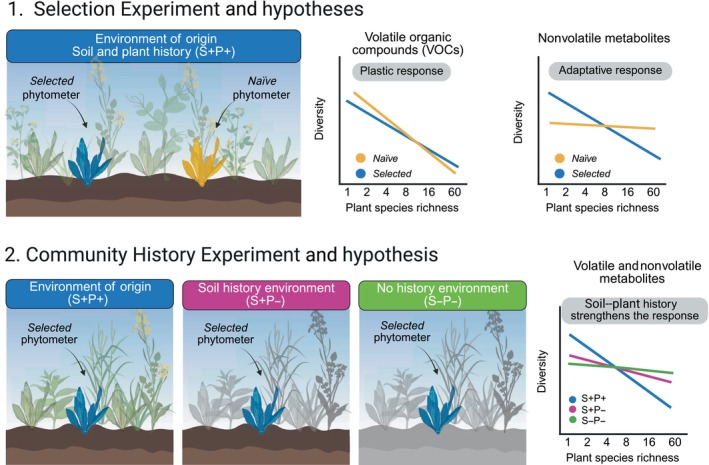
Graphical illustration of the *Selection Experiment* and the *Community History Experiment* with *Plantago lanceolata* phytometers across the plant diversity gradient of the Jena Experiment, Germany. The *Selection Experiment* compared the metabolic profiles between *selected* and *naïve* plants. *Selected* plants (offspring of plants that experienced the selection pressure of environment of origin in the biodiversity environment in the Jena Experiment) were planted in their environment of origin with the *naïve* plants (offspring of plants that did not experience biodiversity selection of the Jena Experiment). The *Community History Experiment* compared the metabolic profiles of *selected* plants grown in different experimental environments based on the Determinants of Long‐Term Biodiversity Effects on Ecosystem Functioning experiment established in 2016 (Vogel *et al*., 2019). *No history* environment (S−P−): soil layer and plant community removed. *Soil history* environment (S+P−): only plant community removed. In both treatments, new plot‐specific plant mixtures were sown in 2016. *Soil–Plant history* environment (S+P+): environment of origin, same as core area established in 2002 (long‐term control). We hypothesized that *P. lanceolata*'s metabolic (both volatile and non‐volatile) diversity will decrease with increasing plant species richness. Volatile diversity will respond plastically, while non‐volatile diversity will show an adaptive response. Community soil and plant history is expected to strengthen these effects. This figure was created in BioRender (BioRender.com/jgxksm8).

Based on the assumption that increased plant species diversity in a community leads to a dilution of antagonists and an accumulation of mutualists (Eisenhauer *et al*., 2024), plants in high‐diversity communities may reduce their investment in both direct and indirect chemical defenses. Consequently, we hypothesized that (H1; Fig. 1) as plant SR increases, both volatile and non‐volatile diversity of *P. lanceolata* will decrease. We further proposed that (H2; Fig. 1) volatile diversity will exhibit a phenotypically plastic response to the immediate surrounding plant SR, as it is largely influenced by (a)biotic interactions within its current environment (Dicke, 2016). Conversely, non‐volatile metabolic diversity will reflect adaptive responses to selection in communities of varying plant SR. Finally, we hypothesized that (H3; Fig. 1) soil–plant community history will strengthen the relationship between plant diversity and the plant metabolic profiles. Removing plant history or soil–plant history is expected to weaken or completely erase the effect of plant SR on both volatile and non‐volatile diversity of *P. lanceolata*.

## Materials and Methods

### Field site

The study was conducted in the Jena Experiment (Jena, Germany; 50°55′N, 11°35′E; 130 m asl), a long‐term grassland biodiversity experiment established in 2002 in Jena, Germany (Roscher *et al*., 2004). The Jena Experiment consists of 82 experimental plots randomly assembled from a species pool of 60 native Central European plant species covering a plant SR gradient from monocultures to 60‐species mixtures (1, 2, 4, 8, 16 and 60 species) and a gradient ranging from 1 to 4 plant functional groups (grasses, small herbs, tall herbs and legumes). The initial plot size of 20 × 20 m was reduced to 6 × 6 m in 2010. To account for natural variation in soil characteristics, the experiment is set up in four blocks containing an equal number of plots per species richness level. For this study, we selected 12 communities (12 plots) in which *Plantago lanceolata* L. (ribwort plantain) belonged to the sown species combinations (Supporting Information Fig. S1), covering a gradient in SR from a *P. lanceolata* monoculture to a 60 plant species mixture (1, 2, 4, 8, 16 and 60 plant species). The study was performed in the Determinants of Long‐Term Biodiversity Effects on Ecosystem Functioning (ΔBEF) Experiment, established inside these communities in 2016 (more details will be discussed later; Vogel *et al*., 2019). All plots were weeded twice per year and managed as mown grasslands (two cuts per year).

### Selection experiment

To explore the effects of experimental selection on *P. lanceolata* metabolic profiles, we used *P. lanceolata* phytometers (= planted individuals) of two seed origins. (1) *Selected seeds:* Seeds collected from four *P. lanceolata* individuals growing in each experimental community with different levels of SR (from *P. lanceolata* monoculture to 60 plant species mixtures), which had experienced the selective pressures of the biodiversity experiment for 17 yr. (2) *Naïve seeds*: Phytometers obtained by growing plants from the initial seed stock used to establish the Jena Experiment in 2002. These plants had no prior exposure to the biodiversity experiment and thus had not experienced selection under those environmental conditions (Fig. 1). Both types of phytometers, *naïve* and *selected*, were grown under controlled conditions as described in De Giorgi *et al*. (2025). When the *selected* phytometers were 10 wk old, they were transplanted into their environment of origin together with *naïve* phytometers (details will be discussed later).

### Community history experiment

To explore the effects of community history on *P. lanceolata* metabolomic profiles, we used *P. lanceolata* phytometers from seeds collected in the 17‐yr‐old communities (the same *selected* seeds used for the *Selection Experiment*) and transplanted them into the ΔBEF treatments of their original community (Fig. 1). The ΔBEF experiment consisted of three subplots (1.5 m × 3 m size) inside the main experimental plots, with different degrees of community history: (1) *Soil and plant history* (S+P+): 17‐yr‐old plant communities (established in 2002), long‐term control (from where the seeds were collected, their environment of origin); (2) *Soil history* (S+P−): Experimental environment in which plant species were removed while keeping the soil and resowing the same species compositions with new seeds; and (3) *No history* (S−P−): Experimental environment in which soil and plant layers were removed and replaced with arable field soil resown with the same species compositions with new seeds. For both treatments, plot‐specific plant mixtures were sown in 2016 using seeds purchased from the same supplier as for the initial establishment of the Jena Experiment in 2002 (Vogel *et al*., 2019).

### Preparation and establishment of phytometer plants

During summer 2018, *P. lanceolata* plants were obtained from the germination of seeds originally used to establish the Jena Experiment, which had been stored at −20°C since 2002. These seedlings were grown in a glasshouse with standard substrate, then transplanted to a seedbed outdoors in autumn at the Experimental Field Station Bad Lauchstädt (Germany). A year later (in summer 2019), seeds from these plants were collected and called *naïve* seeds. *Selected* seeds were collected in 2019 from four mothers of *P. lanceolata* from each 17‐yr‐old community (with soil and plant history, environment of origin), cleaned, and stored at room temperature until the start of the experiment. In January 2020, single seeds (*selected* and *naïve*) were germinated in cells of QuickPot trays (Hermann Meyer KG, Rellingen, Germany) filled with autoclaved soil from the field site mixed with sterile mineral sand (25 vol%) in a glasshouse (temperature of 18°C: 12°C with 14 h of daylight). After 8 wk, the trays were moved into an open glasshouse with outside light and temperature conditions for 2 wk to harden the plants before being planted in the field. In early April 2020, when the plants were 10 wk old, *selected* phytometers were transplanted into the same communities in which the seeds were initially collected (17‐yr‐old communities), while the *naïve* ones were transplanted into all of the plots. In each community, 48 phytometers were transplanted: 12 *naïve* and 12 *selected* phytometers in the environment of origin, 12 *selected* phytometers in the soil history environment, and 12 *selected* phytometers in the no history environment. In total, 567 phytometers were transplanted (De Giorgi *et al*., 2025).

### Sampling and measurements

One year after the transplanting (August 2021), we simultaneously measured morphological and chemical traits of the phytometers of both experiments in the field. These included the collection of headspace volatile emissions, assessment of phenotypic traits, analysis of non‐volatile leaf metabolites, and calculation of percent leaf damage. In six individuals, morphological traits and non‐volatile leaf metabolites were measured. In four of these individuals, headspace volatile collections were performed, and percent leaf damage was assessed.

### Morphological traits and leaf damage

For morphological traits, we recorded the reproductive status, leaf biomass, leaf length, and leaf greenness. Leaf greenness was measured using a portable Chl meter (SPAD 502 Plus, Lonoca Minolta) according to De Giorgi *et al*. (2025). Leaf damage by herbivores and pathogens was assessed using the method described in Unsicker & Mody (2005). Briefly, original leaf areas were reconstructed in digital photographs taken of both leaf sides after the harvest using Adobe Photoshop 2020 (Adobe, CA, USA). Damage was quantified as a percentage of the total leaf area (cm^2^).

### Headspace volatile collection

VOC emitted by *P. lanceolata* phytometers were collected and measured using the protocol described in Medina‐van Berkum *et al*. (2024) with few modifications. In brief, VOC emission of individual plants was captured using a closed push‐pull system over a two‐hour period. The plants were enclosed in PET bags (Polyethylene terephthalate, Bratschlauch, Toppits, Germany), and charcoal‐filtered air was continuously pumped into these bags at a flow rate of 1 l min^−1^. VOC traps, consisting of 25 mg of Porapak absorbent (ARS, Grainville, FL, USA) inserted in Teflon tubes, were attached to the bags, and air was pumped out through them at a flow rate of 0.6 l min^−1^. All volatile collections were performed between 09:00 and 13:00 h over a period of six consecutive days. After collection, the traps were eluted with 200 μl of dichloromethane containing nonyl acetate (Sigma‐Aldrich, 10 ng μl^−1^) as an internal standard. The eluted VOCs were analyzed using an Agilent (Santa Clara, CA, USA) 6890 series gas chromatograph (GC) coupled to either an Agilent 5973 series mass spectrometer (MS) for identification or to a flame ionization detector (FID) for quantification (Medina‐van Berkum *et al*., 2024). VOC identification was achieved by comparing GC‐MS spectra with reference spectra from the Wiley and National Institute of Standards and Technology libraries, as well as by comparing retention times and mass spectra to those of standards from our collection. VOC quantification was determined from GC‐FID data based on the peak area in relation to the peak area of the internal standard. The relative response factor was computed with authentic standards or estimated with the effective carbon number concept and normalized to leaf fresh weight (FW) and duration of collection (nanogram g^–1^ FW h^–1^).

### Metabolite extraction from leaves

Leaf samples were flash frozen in liquid nitrogen after the harvesting, lyophilized, and ground to fine powder by agitating them together with a mix of stainless steel balls (2–4 mm in diameter) in a paint shaker. Then, 10 mg of leaf powder was extracted with 100% methanol (0.1 ml mg^–1^) containing D6‐salicylic acid (SA), D6‐jasmonic acid (JA) and D6‐abscisic acid (ABA) as internal standards (Sigma‐Aldrich). Aliquots of raw extracts were used for: untargeted metabolite profiling; and targeted analysis of phytohormones, iridoid glycosides, and phenylpropanoid glycosides.

### Metabolome profiling

Untargeted metabolic profiles of leaves were obtained by ultra‐high performance liquid chromatography coupled via electrospray ionization (ESI) to a quadrupole time‐of‐flight mass spectrometer (UHPLC‐ESI‐HRMS) in negative ionization mode. The mobile phase consisted of 0.1% v/v formic acid in water and in acetonitrile. Raw data files from UHPLC‐HRMS were transferred to the Metaboscape® (Bruker Daltonics, Hamburg, Germany) software to perform the bucketing based on MS1 spectra. Quality control samples were prepared by pipetting equal volumes of all the samples into a designated LC‐MS vial for analysis and run every 40 samples together with the blanks. Raw data acquisition was carried out as previously described by Medina‐van Berkum *et al*. (2024). The processed LC‐MS/MS data were then used for the *in silico* prediction of chemical taxonomic classification using the Canopus package (Dührkop *et al*., 2021) from the Sirius software (Dührkop *et al*., 2019), considering only classified features with a probability of at least 70% at the pathway level.

### Quantification of targeted compounds

Quantification of targeted compounds was conducted using an HPLC–MS/MS system (HPLC 1260 Infinity II (Agilent Technologies, Santa Clara, CA, USA) – QTrap® 6500+ (AB Sciex, Waltham, MA, USA)) in multiple reaction monitoring mode, following Medina‐van Berkum *et al*. (2024) with few modifications. Phytohormones were quantified with authentic standards (D6‐JA, D6‐ABA and D6‐SA). For iridoid glycosides and verbascoside, quantification was based on comparison to external authentic standard curves (aucubin: Carl Roth, Germany; catalpol: Wako, Japan; verbascoside: Extrasynthese, France).

### Data analysis

We performed mixed‐model analysis and linear discriminant analysis to test the effect of both selection history and community history on leaf traits, leaf damage, and leaf metabolome of *P. lanceolata*. The data from the two experiments, namely the *Selection Experiment* and the *Community History Experiment* were analyzed separately. Leaf metabolome diversity for both volatile and non‐volatile compounds was calculated based on Hill numbers using VOC concentration and peak intensity of features as abundance. To test the effect of biodiversity selection in the *Selection Experiment*, we fitted ‘species richness’ (SR; log_2_‐transformed sown diversity), ‘selection’ (S; factor with two levels: *selected* vs *naïve*) and their interactions (SR × S) as fixed effects. Plot identity nested in block was fitted as random effect. To test the effect of community history in the *Community History Experiment*, we performed a similar model with environment instead of selection as fixed effect (E; factor with three levels: S−P− no history, S+P− with soil history only, and S+P+ soil and plant history). We started with a null model with the random effects only, and successively added the fixed effects with SR first, followed by treatment (selection or environment) and interactions. To investigate the presence of legumes in shaping the selection and community history effect, we created another model by fitting legumes (presence/absence) before or after SR. Since previous analyses have shown that vegetation height varies either with sown SR or depending on environment history (De Giorgi *et al*., 2025), this could potentially explain the effects of both factors. Therefore, we performed another model in which we included the mean height of the surrounding vegetation as a covariable fitted before the experimental factors. All models were fitted with maximum likelihood (ML), and Wald tests were used to decide on the significance of the fixed effects. When needed, data were transformed to meet the assumptions of normality and homogeneity of variances. To identify non‐volatile metabolic features significantly affected by the treatments, we used generalized linear mixed models (Gaussian log link) based on the previously mentioned model structures. The significance of fixed effects (*P* < 0.05) was assessed by Wald tests, followed by false discovery rate adjustment for *P*‐values.

The analyses and visualization were conducted in R v.4.3.3 (R Core Team, 2024) using the following packages: rBExIS, dplyr (Wickham *et al*., 2023), Tidyverse (Wickham *et al*., 2019), Tibble (Müller & Wickham, 2023) and Janitor (Firke, 2023) for data retrieval, cleaning and formatting; lme4 (Bates *et al*., 2015), lmerTest (Kuznetsova *et al*., 2017), glmmTMB (Brooks *et al*., 2017), performance (Lüdecke *et al*., 2021), mixOmics (Rohart *et al*., 2017) and hillR (Li, 2018) for statistical and diversity analysis; Notame (Klavus *et al*., 2020) for filtering false‐positive signals of untargeted metabolites; and ggplot2 (Wickham, 2016), ggeffects (Lüdecke, 2018) and pheatmap (Kolde, 2019) for graphical visualization. Graphics were enhanced with Adobe Illustrator CC 2021.

## Results

### Effects of selection and community history on plant performance and leaf damage

**Fig. 2:**
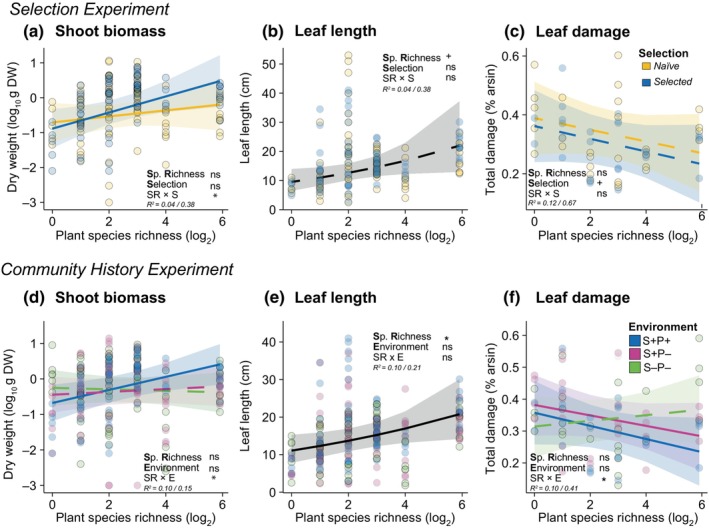
Effects of selection history and community history on leaf traits and leaf damage of *Plantago lanceolata* across a plant diversity gradient. *Selection Experiment* (top section): (a) shoot biomass, (b) leaf length, and (c) total leaf damage (herbivore + pathogen damage) of *naïve* (offspring of the original seed material used in the establishment of the Jena Experiment) and *selected* (offspring of plants that experienced selection pressures in the biodiversity experiment) phytometers across a plant diversity gradient. *Community History Experiment* (bottom section): (d) shoot biomass, (e) leaf length, and (f) total leaf damage of *selected* phytometers grown in three different environments: their environment of origin (blue dots; soil and plant history, S+P+), in the environment with soil history only (S+P−) or no history environment (S−P−) across a plant diversity gradient. The different environment treatments are based on the Determinants of Long‐Term Biodiversity Effects on Ecosystem Functioning experiment established in 2016 (Vogel *et al*., 2019). Lines represent predictions from linear mixed‐effects models. Solid lines denote significant species richness relationships (*P* < 0.05) and dashed lines show nonsignificant relationships. Points represent each phytometer. Asterisks indicate significant effects (ns, no significant; +, *P* < 0.1; *, *P* < 0.05) on species richness (SR), selection history (S), community history (E) or their interactions (SR × S or SR × E). Marginal and conditional *R* ^2^ (marginal before slash and conditional after) are reported. *Selection Experiment*: Sample size = 112 and 56 individuals for leaf traits and leaf damage, respectively. *Community History Experiment*: Sample size = 169 and 80 individuals for leaf traits and leaf damage, respectively.

We found that only *selected* phytometers (offspring of plants that experienced the selective pressure of environment of origin in the biodiversity experiment) showed an increase in shoot biomass with increasing SR of the community, while *naïve* phytometers (offspring of plants that did not experience these biodiversity selection pressures) had similar shoot biomass across the diversity gradient (SR × S: *x* ^2^ = 4.95, *P* = 0.026; Fig. 2a; Table S1). This pattern was only true when *selected* phytometers grew in their environment of origin (SR × E: *x* ^2^ = 9.86, *P* = 0.007; Fig. 2d; Table S2) and not when community history had been eliminated by removing surrounding soil and plants or plants alone. Leaf greenness and leaf length did not differ between *naïve* and *selected* phytometers (Fig. 2b; Table S1). However, phytometers increased their leaf greenness when they grew in communities with legumes compared with nonlegumes communities (*x* ^2^ = 9.11, *P* = 0.003; Table S1). In the *Community History Experiment*, we found that leaf length increased with increasing SR regardless of the environment treatment (*x* ^2^ = 4.71, *P* = 0.03, Fig. 2e). Similar to the *Selection Experiment* results, the presence of legumes in the plot enhanced leaf greenness in *P. lanceolata* (*x* ^2^ = 13.68, *P* < 0.001; Table S2) while it decreased with increasing vegetation height in their surroundings (*x* ^2^ = 7.56, *P* = 0.006; Table S2).

*Naïve* phytometers had a tendency to experience higher leaf damage than the *selected* ones (*x* ^2^ = 2.77, *P* = 0.096; Fig. 2d). When phytometers grew in the environment with no plant–soil history (S−P−), leaf damage increased with increasing SR (SR × E: *x* ^2^ = 6.19, *P* = 0.045; *post hoc* S−P− vs S−P+ and S+P+: < 0.05; Fig. 2h). These patterns were primarily driven by changes in pathogen damage along the plant SR gradient rather than herbivory damage (Table S2). Additionally, an increase in vegetation height in the surroundings reduced leaf pathogen damage of *P. lanceolata* phytometers (Table S1).

### Volatile and non‐volatile leaf metabolic profiles

A total of 31 VOCs were identified from the headspace volatile collection of *P. lanceolata* in the field (Table S3). These VOCs were categorized into green leaf volatiles (GLVs) (5), aromatics (4), homoterpenes (1), monoterpenes (6), sesquiterpenes (8) and nine other compounds not classified into these groups. Sesquiterpenes represented the most diverse group, while monoterpenes and GLVs were the most abundant.

**Fig. 3:**
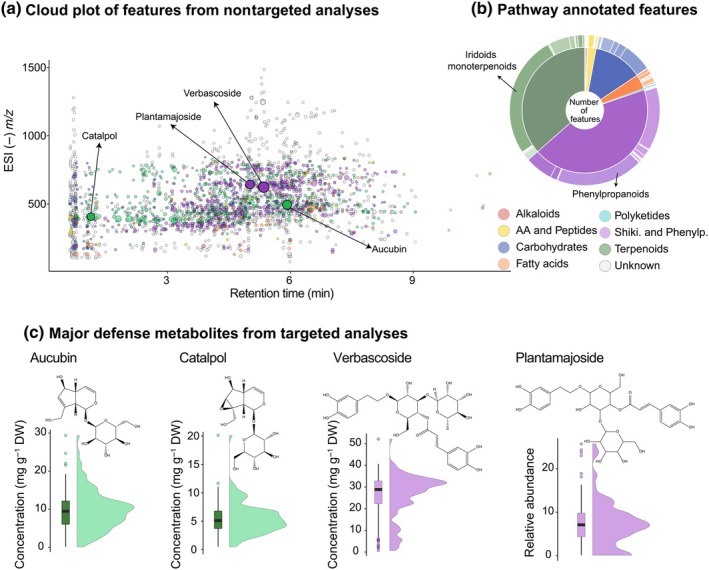
Profiles of non‐volatile leaf metabolites of *Plantago lanceolata* phytometers 1 yr after transplanting in the Jena Experiment. (a) Cloud plot of metabolic features from untargeted LC‐MS measurements made in the negative ionization mode. Features are color‐coded based on putative biosynthetic pathway classification; the size of the circles represents their intensity; (b) Pie chart with the number of features classified by biosynthetic pathway. A total of 2263 were detected, with 49% of the features putatively annotated. Biosynthetic pathway classification was performed with CANOPUS on Sirius platform. (c) Boxplot and violin plots of concentrations of aucubin, catalpol, verbascoside and relative abundance of plantamajoside in the leaves of all phytometers measured by targeted LC‐MS analysis. Boxplots show the median (horizontal line), interquartile range (box limits = 25^th^ to 75^th^ percentiles) and whiskers extending to the most extreme values within 1.5 times the interquartile range. Individual points outside this range are plotted as outliers. Violin plots overlay kernel density estimates to illustrate the full distribution of the data.

Overall, we detected 2263 features in leaf extracts of non‐volatile compounds from *P. lanceolata* analyzed by untargeted LC‐MS measurements in the negative ionization mode, with 49% of the features putatively annotated by CANOPUS. Based on these *in silico* classification, the terpenoid and shikimate–phenylpropanoid pathways were the most dominant pathways in the leaf metabolome of *P. lanceolata* (Fig. 3a, b). Iridoid monoterpenoids constituted the most abundant class (72%) within terpenoids, largely due to the high number of iridoid glycosides, such as aucubin and catalpol, which are two of the most abundant examples, reaching up to 30 mg g^–1^ dry weight (DW) based on targeted analyses (Fig. 3c). Phenylpropanoids comprised 42% of the metabolic features and were thus the most diverse class within the shikimates and phenylpropanoids pathway. Verbascoside and plantamajoside are two of the most abundant phenylpropanoids in *P. lanceolata* leaves, reaching up to 50 mg g^–1^ DW (Fig. 3c).

### Plant metabolic profile responses to selection history

**Fig. 4:**
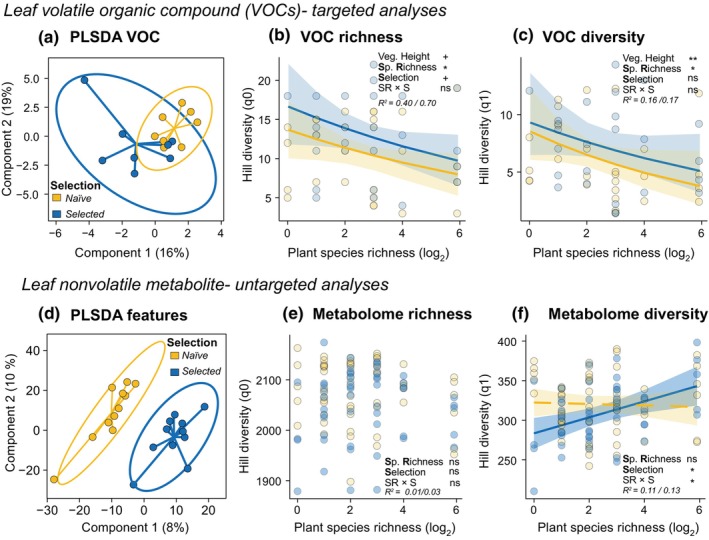
*Selection Experiment*: Effects of selection history on leaf metabolite profiles of *Plantago lanceolata* across a plant diversity gradient. *Leaf* volatile organic compounds (*VOCs*) (top section): Overall, a total of 31 VOCs were identified. (a) Partial least square discriminant analysis (PLS‐DA) of the VOC profile in *naïve* (offspring of the original seed material used for the establishment of the Jena Experiment) and *selected* (offspring of plants that underwent the selection pressures in the biodiversity experiment) phytometers. The results are presented as principal component score plots, with each point in the plot representing a mean value of phytometers in a community; (b) VOC richness (Hill richness – q0) and (c) VOC diversity (Hill Shannon – q1) across a plant richness gradient. *Leaf non‐volatile metabolites* (bottom section): There were 2263 metabolic features in the nontargeted analysis in the negative ionization mode after bucketing and filtering. (d) PLS‐DA of metabolic features, (e) Metabolome richness (Hill richness – q0), (f) Metabolome diversity (Hill Shannon – q1) of *naïve* and *selected* phytometers across a plant diversity gradient. Lines represent predictions from linear mixed‐effects models. Solid lines denote a significant species richness relationship (*P* < 0.05), and dashed lines show a nonsignificant relationship. Points represent each phytometer. Asterisks indicate significant effects (ns, no significant; +, *P* < 0.1; *, *P* < 0.05; **, *P* < 0.01) on species richness (SR), selection history (S) or their interaction (SR *×* S). Marginal and conditional *R* ^2^ (marginal before slash and conditional after) are reported. Sample size = 60 and 108 individuals for VOC and metabolic feature profiles, respectively.

Overall, VOC profiles overlapped between *naïve* and *selected P. lanceolata* phytometers (Fig. 4a). Total emission was unaffected by plant SR or selection history (Table S4). Nevertheless, when considering the vegetation height of the surroundings, sesquiterpene emission decreased as plant SR in the community increased (*x* ^2^ = 6.93, *P* = 0.008; Table S4). Accounting for vegetation height also revealed that both VOC richness (Hill q0) and diversity (Hill q1) decreased with increasing plant SR in the community (richness: *x* ^2^ = 4.72, *P* = 0.030; diversity: *x* ^2^ = 3.97, *P* = 0.048; Fig. 4b, c). *Selected* phytometers tended to exhibit higher numbers of VOCs (Hill q0) than *naïve* phytometers (*x* ^2^ = 3.20, *P* = 0.074; Fig. 4b).

In terms of non‐volatile compounds, *naïve* and *selected P. lanceolata* phytometers displayed distinctly different metabolic profiles (Fig. 4d). The number of metabolic features was not influenced by SR (*x* ^2^ = 0.19, *P* = 0.662; Fig. 4e); however, considering feature intensity (Hill q1 and q2), metabolite diversity increased with increasing SR in *selected* phytometers, while the metabolite diversity of *naïve* phytometers was not affected by plant SR (SR × S: Hill q1: *x* ^2^ = 6.35, *P* = 0.012; Hill q2: *x* ^2^ = 7.90, *P* = 0.005; Fig. 4f). Vegetation height in their surroundings did not influence the overall metabolite richness and diversity of *P. lanceolata* individuals (Table S5).

Overall, 689 metabolic features in *P. lanceolata* leaf extracts differed significantly in their intensity across the diversity gradient, depending on selection history or their interaction (34% of the whole metabolome), including a mixture of features from different metabolic classes, mainly terpenoids and shikimates/phenylpropanoids (Fig. S2). SR had an impact on 224 unique features (10% of the whole metabolome), of which 65% decreased in intensity with increasing SR (Fig. S2). Selection history affected 18% of the metabolome, in which the majority of features had higher intensity in *naïve* phytometers than in *selected* phytometers. Furthermore, 9% of the features were affected by the interaction between SR and selection history, suggesting that these features in *selected* phytometers reacted differently to SR than they did in *naïve* phytometers. The increased vegetation height of the surrounding plant community in high‐diversity mixtures influenced 11% of the metabolome. Additionally, the presence of legumes influenced the feature intensity in response to SR and selection, both positively and negatively (Fig. S2).

Based on these results, we quantified some of the main defense hormones in *P. lanceolata* and the major antiherbivore defense compounds, the iridoid glycosides aucubin and catalpol and the phenylpropanoid glycosides verbascoside and plantamajoside (Fig. 2; Tables 1, S6). Considering the vegetation height of the surrounding plant community, we found that the concentration of aucubin, verbascoside, and plantamajoside decreased with increasing SR regardless of the selection history (aucubin: *x* ^2^ = 9.34, *P* = 0.002; verbascoside: *x* ^2^ = 4.65, *P* = 0.031; plantamajoside: *x* ^2^ = 4.96, *P* = 0.026). The negative relationship of verbascoside foliar concentration with SR was stronger in communities with legumes than in the ones without legumes. Aucubin concentrations increased with increasing vegetation height of the surrounding plant community (*x* ^2^ = 8.12, *P* = 0.004; Table 1). For defensive hormones, SR influenced SA and ABA concentrations, although they did not affect the levels of jasmonates overall (Table 1; see Table S6, for specific patterns of each jasmonate). The concentration of SA and ABA decreased with increasing SR (SA: *x* ^2^ = 7.60, *P* = 0.005; ABA: *x* ^2^ = 3.54, *P* = 0.048). However, the decrease in ABA concentrations was driven by the increasing vegetation height of the surrounding plant community (*x* ^2^ = 9.35, *P* = 0.002; Table 1).

**Table 1:** *Selection Experiment*: Wald chi‐squared analysis of variance results for the linear mixed models of *Plantago lanceolata* hormones and other non‐volatile defense compounds quantified by targeted analyses.

Variable	Model	*R* ^2^	Vegetation height (VG)	Species richness (SR)	Legumes (L)	Selection (S)	SR × L	SR × S	L × S	SR × L × S
Defense hormones										
Jasmonates *log*_*10*_ *n* = 112	y~SR*S	0.05/na	na	0.94	na	1.37	na	0.40	na	na
	y~SR*L*S	0.13/na	na	0.94	0.10	1.34	2.72+	0.43	0.83	0.89
	y~VG + SR*L*S	0.14/na	0.89	0.12	0.50	1.52	2.10	0.44	0.83	0.88
Abscisic acid *log*_*10*_ *n* = 112	y~SR*S	0.11/na	na	3.54*↓	na	0.06	na	1.52	na	na
	y~SR*L*S	0.15/0.25	na	3.54*↓	0.05	0.07	3.00+	1.52	0.71	1.44
	y~VG + SR*L*S	0.3/na	9.35**↓	0.13	2.44	0.12	1.41	1.69	0.64	1.43
Salicylic acid *log1p n* = 112	y~SR*S	0.26/0.43	na	7.06*↓	na	1.64	na	3.52+	na	na
	y~SR*L*S	0.32/0.45	na	7.06*↓	0.82	1.53	0.46	3.54+	0.14	0.19
	y~VG + SR*L*S	0.35/0.46	3.29 + ↑	6.05*↓	2.80+	0.01	0.97	2.10	0.04	1.56
Iridoid glycosides										
Aucubin^1^ *n* = 107	y~SR*S	0.02/0.13	na	0.15	na	0.81	na	1.04	na	na
	y~SR*L*S	0.09/0.15	na	0.15	1.3	0.99	2.48	0.96	0.04	3.51+
	y~VG + SR*L*S	0.23/na	8.12**↑	9.34**↓	1.01	1.19	0.79	1.06	0.06	3.69+
Catalpol^1^ *n* = 107	y~SR*S	0.02/na	na	0.29	na	0.00	na	0.93	na	na
	y~SR*L*S	0.07/na	na	0.29	0.04	0.00	2.85+	0.81	0.59	0.45
	y~VG + SR*L*S	0.11/na	2.52	0.12	2.08	0.00	2.9+	0.73	0.41	0.29
Phenylpropanoid glycosides										
Verbascoside^1^ *n* = 107	y~SR*S	0.1/na	na	2.61	na	0.88	na	0.05	na	na
	y~SR*L*S	0.25/0.34	na	2.61	0.59	0.97	4.4*↓	0.07	0.06	4.03*
	y~VG + SR*L*S	0.28/na	0.04	4.59*↓	0.00	1.04	3.92*↓	0.07	0.05	3.99*
Plantamajoside *sqrt n* = 112	y~SR*S	0.12/na	na	7.74**↓	na	0.34	na	0.15	na	na
	y~SR*L*S	0.2/na	na	7.74**↓	0.35	0.39	1.21	0.19	0.97	2.86+
	y~VG + SR*L*S	0.2/na	2.70	5.17*↓	0.79	0.37	0.82	0.19	1.00	2.84+

The table reports marginal and conditional *R* ^2^ (marginal before slash and conditional after), the number of samples and *x* ^2^‐values for each model (rows). na indicates that the fixed factor was not included in the model. Level of significance is based on *P*‐values and reported with asterisks and dots: ***, *P* < 0.001; **, *P* < 0.01; *, *P* < 0.05; +, *P* < 0.1. Arrows next to the *x* ^2^‐values indicate the patterns: increase (↑) or decrease (↓) in relation to the fixed factor (column).

^1^ Five samples were lost during the LC‐MS quantification of the compounds.

### Plant metabolic profile responses to community history

**Fig. 5:**
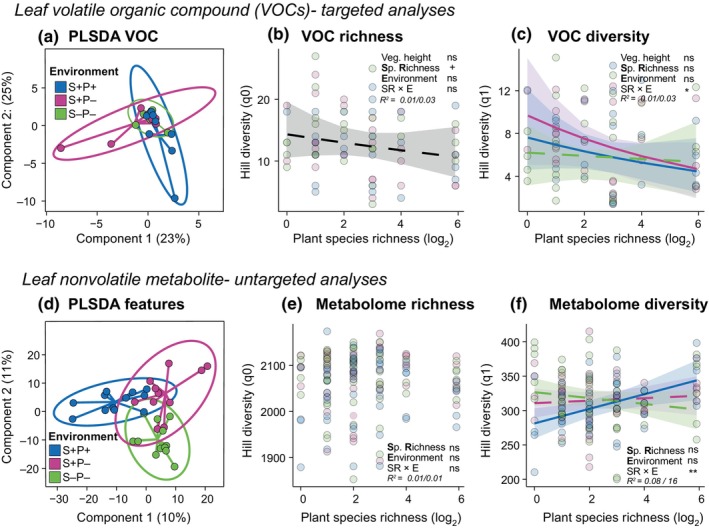
*Community History Experiment*: Effects of community history on volatile and non‐volatile leaf metabolites of *Plantago lanceolata* across a plant diversity gradient. *Leaf* volatile organic compounds (*VOCs*) (top section): Overall, a total of 31 VOCs were identified. (a) Partial least square discriminant analysis (PLS‐DA) of VOCs from *selected* phytometers that grew in their environment of origin (soil–plant history, S+P+), in the environment with soil history (S+P−) or no history environment (S−P−). The results are presented as principal component score plots, with each point in the plot representing a mean value of phytometers in a community; (b) VOC richness (Hill richness – q0) and (c) VOC diversity (Hill Shannon – q1) across a plant richness gradient. *Leaf non‐volatile metabolites* (bottom section): There were 2263 metabolic features in the negative ionization mode after bucketing and filtering. (d) PLS‐DA of the metabolites of *selected* phytometers that grew in their environment of origin, in the environment with soil history or no history environment. The results are presented as principal component score plots, with each point in the plot representing a plot, (e) Metabolome richness (Hill richness – q0), and (f) Metabolome diversity (Hill Shannon – q1) across a plant richness gradient. Lines represent predictions from linear mixed‐effect models. Solid lines denote a significant species richness relationship (*P* < 0.05), and dashed lines show a nonsignificant relationship. Points represent each phytometer. Asterisks indicate significant effects (ns, no significant; +, *P* < 0.1; *, *P* < 0.05; **, *P* < 0.01) on species richness (SR), community history (E) or their interaction (SR × E). Marginal and conditional *R* ^2^ (marginal before slash and conditional after) are reported. Sample size = 87 and 156 individuals for VOC and metabolic feature profiles, respectively.

VOC profiles of *selected P. lanceolata* did not vary among the different environments (Fig. 5a). Nevertheless, phytometers in environments without history (S−P−) displayed greater similarity in VOC profiles across the SR gradient than those in environments with soil history (S+P+, S+P−). While total emission was not influenced by environment, monoterpene emission decreased with increasing SR only in environments with soil history (S+P+, S+P−: SR × E: *x* ^2^ = 8.53, *P* = 0.014; *post hoc* < 0.05; Table S7). Emission of sesquiterpene and aromatic compounds decreased with SR when considering the surrounding vegetation height (sesquiterpenes: *x* ^2^ = 6.84, *P* = 0.008; aromatics: *x* ^2^ = 4.68, *P* = 0.031; Table S7). Neither SR nor community history directly influenced the number of VOCs (Hill q0) in *P. lanceolata* (Fig. 5b). However, accounting for vegetation height revealed that VOC diversity (Hill q1) decreased with increasing SR only in phytometers that grew in environments with soil history (S+P+, S+P−), but remained similar across the SR gradient in environments without soil–plant history (S−P−; SR × E: *x* ^2^ = 5.13, *P* = 0.048; Fig. 5c).

The non‐volatile leaf metabolic composition of *selected P. lanceolata* phytometers was influenced by the experimental environment in which they grew (Fig. 5d). The number of non‐volatile metabolic features did not differ among environment treatments (*x* ^2^ = 0.42, *P* = 0.809; Fig. 5e). However, when considering the intensity of these features, we found a significant interaction between SR and community history. Specifically, metabolic diversity increased with increasing SR only in phytometers growing in environments of origin (S+P+), whereas for phytometers in the other experimental environments (S+P−, S−P−), the response to SR was weaker (Hill q1: *x* ^2^ = 10.28, *P* = 0.006; Hill q2: *x* ^2^ = 8.19, *P* = 0.017; *post hoc* < 0.01; Fig. 5f; Table S8). Additionally, the surrounding vegetation height did not influence the overall metabolome richness and diversity of *P. lanceolata* phytometers (Table S8).

Overall, 830 features significantly differed in intensity among the experimental environments (37% of the whole metabolome). Phytometers growing in the environment of origin had lower intensity for most of their features compared with phytometers growing in the other experimental environments (Fig. S3). SR had an impact on 374 features (16% of the whole metabolome), of which 52% decreased in intensity with increasing SR (Fig. S4). Regarding the impact of community history (16% of the features), we discovered that most features intensified when phytometers grew in experimental environments without history (S−P−, S+P−). Moreover, 12% of the features showed an interaction effect between SR and experimental environment, primarily due to different strengths in responses of phytometers in the environment with soil–plant history (S+P+) and no history (S−P−; Fig. S4).

While foliar concentration of catalpol did not change across the SR gradient, aucubin concentration decreased as plant SR increased when vegetation height in the surroundings was taken into consideration (*x* ^2^ = 6.32, *P* = 0.012). Catalpol concentration was higher in environments with soil and plant history (*x* ^2^ = 6.27, *P* = 0.043); however, this effect became a tendency when we considered the vegetation height of the surroundings (*x* ^2^ = 9.77, *P* = 0.002). Considering the presence of legumes, verbascoside concentration increased with SR in the presence of legumes but decreased in communities without legumes, regardless of the experimental environment (*x* ^2^ = 9.47, *P* = 0.002; Table 2). The relative abundance of plantamajoside decreased with SR only in communities with legumes (*x* ^2^ = 9.07, *P* = 0.002; Tables 2, S9).

**Table 2:** *Community History Experiment*: Wald chi‐squared analysis of variance results for the linear mixed models of *Plantago lanceolata* plant defense hormones and other non‐volatile defense compounds quantified by targeted analyses.

Compounds	Model	$R_{m/c}^{2}$	Vegetation height (VG)	Species richness (SR)	Legumes (L)	Environment (E)	SR × L	SR × E	L × E	SR × L × E
Defense hormones										
Jasmonates *log*_*10*_ *n* = 163	y~SR*E	0.05/0.28	na	0.83	na	1.87	na	4.00	na	na
	y~SR*L*E	0.14/0.27	na	0.83	0.11	1.84	4.45*↓	4.03	1.75	2.09
	y~VG + SR*L*E	0.19/na	2.27	2.32	0.37	0.93	3.71+	3.45	1.18	2.36
Abscisic acid *log*_*10*_ *n* = 163	y~SR*E	0.07/na	na	1.36	na	1.12	na	5.94+	na	na
	y~SR*L*E	0.1/na	na	1.36	0.03	1.12	2.20	5.99+	0.38	0.27
	y~VG + SR*L*E	0.11/na	0.58	0.85	0.01	1.28	2.44	6.38*↓	0.35	0.53
Salicylic acid *log*_*10*_ *n* = 163	y R*E	0.02/na	na	5.59*↓	na	1.81	na	0.79	na	na
	y~SR*L*E	0.02/na	na	5.59*↓	0.01	1.82	8.82**↓	0.91	1.21	10.62**↑
	y~VG + SR*L*E	0.02/na	3.43+↑	3.56+	0.23	1.3	10.46**↓	1.17	1.46	10.89**↑
Iridoid glycosides										
Aucubin *n* = 163	y~SR*E	0.03/0.12	na	0.94	na	1.94	na	0.55	na	na
	y~SR*L*E	0.06/0.14	na	0.94	1.98	1.84	0.04	0.50	0.12	3.90
	y~VG + SR*L*E	0.13/na	3.56+↑	6.32*↓	0.11	2.63	0.00	0.31	0.30	3.75
Catalpol *n* = 163	y~SR*E	0.07/0.12	na	1.19	na	6.27*↑	na	2.54	na	na
	y~SR*L*E	0.1/0.16	na	1.19	0.43	6.26*↑	0.54	2.51	0.46	4.07
	y~VG + SR*L*E	0.17/na	9.77**↑	0.58	4.14*	4.84+	0.60	1.82	0.77	2.76
Phenylpropanoid glycosides										
Verbascoside *n* = 163	y~SR*E	0.06/na	na	0.61	na	2.77	na	3.85	na	na
	y~SR*L*E	0.25/0.33	na	0.61	0.47	2.73	8.22**↓	3.72	1.03	5.24+
	y~VG + SR*L*E	0.28/na	0.05	0.97	0.28	2.00	10.04**↓	3.30	0.90	6.47*↓
Plantamajoside *n* = 163	y~SR*E	0.05/na	na	0.67	na	2.99	na	3.43	na	na
	y~SR*L*E	0.28/na	na	0.67	0.00	2.99	9.07**↓	3.54	1.91	11.04**↓
	y~VG + SR*L*E	0.28/na	1.11	0.17	0.08	2.58	9.07**↓	3.69	1.89	10.65**↓

The table reports marginal and conditional *R* ^2^ (marginal before slash and conditional after), the number of samples and *x* ^2^‐values for each model (rows). na indicates that the fixed factor was not included in the model. Level of significance is based on *P*‐values and reported with asterisks and dots. ***, *P* < 0.001; **, *P* < 0.01; *, *P* < 0.05; +, *P* < 0.1. Arrows next to the *x* ^2^‐values indicate the patterns: increase (↑) or decrease (↓) in relation to the fixed factor (column).

Considering the defense hormones, we observed that jasmonates concentration, in most cases, remained unaffected by SR or experimental environment (Tables 2, S9), while SA decreased with increasing SR (*x* ^2^ = 5.59, *P* = 0.018; Table 2). ABA decreased with increasing SR only in *P. lanceolata* phytometers in environments with soil and plant history when considering the surrounding vegetation height (S+P+; *x* ^2^ = 6.38, *P* = 0.041; Table 2). Considering the presence of legumes in the model, SA concentration decreased with increasing SR in nonlegume communities. In plots with legumes, SA only increased with SR in the environment with no history (S−P−), whereas in other environments, there was no effect of SR (Table 2).

## Discussion

Selection pressures from interspecific interactions between plants and other organisms may select for plants with traits that promote coexistence. In a recent study, De Giorgi *et al*. (2025) demonstrated that selection history plays a crucial role in enhancing the performance of several grassland species within high‐diversity plant communities. To explore whether these selection effects extend to metabolic diversity, we focused on *P. lanceolata* phytometers and examined their metabolic changes, both volatile and non‐volatile compounds, across a plant diversity gradient. We found that volatile diversity of *P. lanceolata* decreased with increasing plant SR in the surrounding community, while their non‐volatile metabolic diversity increased. However, volatile diversity responded to the increase of plant SR diversity through plasticity rather than selection. Moreover, soil history enhanced the decrease of VOC diversity with SR. By contrast, non‐volatile diversity only increased with increasing plant SR in individuals that underwent plant diversity‐driven selection. The effects were more pronounced when plants shared soil–plant history with their community. In summary, our findings indicate that 17 yr of selection history in the biodiversity experiment induced both plastic and adaptive responses in the metabolome of *P. lanceolata* in relation to plant species diversity with these effects strengthening over time as the soil and plant community aged.

### Plant diversity affects the composition and diversity of leaf metabolome

Earlier studies have shown that grassland species displayed metabolic variation in response to the diversity or plant identity of their surrounding community (Scherling *et al*., 2010; Kigathi *et al*., 2013, 2019). Similar results have been observed in *P. lanceolata*, in which SR directly and indirectly influenced the foliar concentration of major defense compounds (Mraja *et al*., 2011). Our results align with these studies, demonstrating that both volatile and non‐volatile compounds in leaves responded to increasing plant diversity in their surroundings. Interestingly, the response of these compounds exhibited opposing patterns: Volatile diversity decreased with increasing SR in the community, while non‐volatile diversity increased (Fig. 4).

A key driver of the plant's metabolome is antagonistic pressure, which can vary depending on the surrounding plant community. Low‐diversity plant communities tend to accumulate and be dominated by plant antagonists above‐ and belowground (Thakur *et al*., 2021). Previous studies have shown that *P. lanceolata* plants experienced higher leaf damage in low‐diversity plant communities than in high‐diversity plant communities (Lipowsky *et al*., 2011), although some studies found no effect of plant diversity (Mraja *et al*., 2011). In our study, we did not observe a reduction in herbivory damage in plants growing in high‐diversity plant communities; instead, with increasing diversity, we observed a reduction in leaf pathogen damage, which followed a similar pattern to SA concentration, although there was no significant correlation between them. Plant pathogens are typically categorized into biotrophs and necrotrophs based on their lifestyles. SA is usually induced upon biotrophic leaf pathogens, while JA‐ and ethylene‐depended responses are triggered by necrotrophic pathogens (Glazebrook, 2005). The lack of significant correlation between SA or JA levels and leaf pathogen damage was expected. This is likely because phytohormone levels reflect short‐term responses, while pathogen damage accumulated over the survey period.

An increase in community plant SR leads also to alterations in light and nutrient availability, competition with neighbors, and herbivore or pathogen load (Roscher *et al*., 2008; Ebeling *et al*., 2014; Rottstock *et al*., 2014; Bachmann *et al*., 2018; Kigathi *et al*., 2019). Light availability can strongly modulate the biosynthesis of compounds in plants through a range of mechanisms, such as photosynthesis, light wavelength, photoperiod, and carbon and nitrogen allocations (Liu *et al*., 2023). Moreover, the presence of N_2_‐fixing legumes in the community enhances soil nitrogen availability (Hartwig, 1998; Roscher *et al*., 2011). In *P. lanceolata*, light and nutrient availability have been identified as crucial players driving the variation in main defense compounds (Mraja *et al*., 2011; Miehe‐Steier *et al*., 2015). We observed that VOC diversity decreased with increasing plant diversity when considering the vegetation height of the surrounding community; that is, sesquiterpene emissions were reduced in tall vegetation. Additionally, communities with legumes showed an overall increase in VOC emissions and richness compared with those without legumes. While the overall diversity of the non‐volatile metabolome was not affected by vegetation height, some specific metabolic features were influenced by both vegetation height and the presence of legumes (affecting 10% and 7% of features detected in negative ionization mode, respectively). These findings align with previous research (Scherling *et al*., 2010), indicating that certain metabolic features are sensitive to light and nutrient availability, thereby confirming these factors as key drivers of plant metabolome profiles.

While we found a positive relationship between metabolome diversity and plant SR, we found that this relationship was primarily driven by chemical evenness rather than chemical richness. More specifically, *P. lanceolata* individuals in low‐diversity plant communities showed a decreased emission of VOCs in comparison to high‐diversity plant communities. This led to reduced diversity in both the number of compounds and the emission of VOCs. Conversely, in low‐diversity plant communities, plants exhibited increased intensity of several non‐volatile metabolic features, leading to a reduction in chemical evenness. Dominant defense compounds, such as aucubin and verbascoside, decreased in concentration as plant diversity increased. This observation is consistent with the resource dilution hypothesis (Otway *et al*., 2005), which suggests that plants in high‐diversity plant communities experience reduced herbivore damage and pathogen pressure, leading to decreased investment in defense compounds. This might also be explained by associational effects, in which *P. lanceolata* might benefit from being surrounded by plants with different chemical profiles (Hambäck *et al*., 2014).

Overall, in low‐diversity plant communities, plants emit highly diverse VOC bouquets but display low non‐volatile metabolic diversity, which is driven by high concentrations of major defense compounds. By contrast, in high‐diversity plant communities, plants decrease VOC diversity but increase non‐volatile diversity due to having a more even composition. This suggests a shift in defense strategies between low‐diversity and high‐diversity plant communities. As community diversity increases, plants interact with a broader range of organisms, both within and across species. These interactions involve diverse metabolites that play active roles. The variation in defense strategies and responses to species diversity observed in our experiments aligns with the Interaction Diversity Hypothesis (Wetzel & Whitehead, 2020) or the Common‐Sense Scenario (Berenbaum & Zangerl, 1996). Both perspectives propose that plant chemodiversity is influenced by intricate multispecies interactions, which simultaneously drive and reflect their chemical complexity. Further research is needed to determine whether these differences represent a diversity‐mediated transition from direct to indirect defense strategies.

### *Plantago lanceolata* exhibits both phenotypic plasticity and adaptations at the metabolic level

Plant diversity can create differential selection pressures between low‐diversity and high‐diversity plant communities (Zuppinger‐Dingley *et al*., 2015). These pressures influence plant phenotypes through both plasticity and genetic processes, affecting traits at the morphological and chemical level, which in the end leads to better performance (Defossez *et al*., 2021; Thon *et al*., 2024). *P. lanceolata* performance, measured by total aboveground biomass, was better in *selected* phytometers than in the *naïve* ones in their environment of origin. As a consequence, *naïve* and *selected* phytometers in low‐diversity plant communities had similar biomass, but in high‐diversity plant communities, *selected* phytometers had higher biomass than *naïve* ones. In other words, *selected* phytometers benefit from species‐rich communities.

We hypothesized that if there is a diversity‐inflicted selection pressure at the plant metabolome level, we would observe a relationship between SR and metabolic diversity only in *selected* phytometers, while *naïve* phytometers would display a similar metabolic diversity along a plant diversity gradient. Our results showed metabolic differences between *naïve* and *selected* phytometers growing in the same environment, but these changes were primarily evident in non‐volatile profiles, while volatile profiles were similar between *naïve* and *selected* phytometers. Specifically, volatile diversity decreased with increasing plant SR, regardless of the selection history of the phytometers. On the other hand, non‐volatile diversity showed a positive relationship with plant diversity only in *selected* phytometers. These results suggest that plants exhibit both phenotypic plasticity and adaptations in their leaf metabolome.

Plants emit VOCs constitutively, but most of their VOC diversity originates from induced responses to biotic and abiotic stress. This phenotypic plasticity allows plants to communicate with beneficial organisms (such as predators and parasitoids of insect herbivores) and detrimental ones, and to send messages to other conspecifics and parts of the same plant. To achieve this, plants must be able to recognize and differentiate between different neighbors and adjust their phenotype accordingly (Dicke, 2016). Therefore, it is more likely that plants exhibit a plastic response in VOC emissions rather than an adaptive response, especially since they are essential for intra‐ and interspecific communication within their surroundings. On the other hand, our study revealed that non‐volatile metabolic diversity showed a positive relationship with plant diversity only in *selected* phytometers. Interestingly, when we looked at specific compounds, we found that iridoid glycoside and verbascoside variation in *P. lanceolata* across a diversity gradient appears to be driven primarily by phenotypic plasticity rather than by selection of genotypes better suited to a particular plant community, a pattern reported previously (Miehe‐Steier *et al*., 2015). Although previous studies have demonstrated that the production of iridoid glycosides is heritable (Marak *et al*., 2000), we found no significant differences between *naïve* and *selected* phytometers. This suggests that diversity‐driven selection pressures may have limited influence on their production. Instead, the variation in compound concentrations likely represents a plastic response to the surrounding diversity. Nonetheless, the relative contributions of plasticity and adaptation to these responses remain unclear and merit further investigation.

Natural selection at the metabolome level occurs when metabolites that provide benefits become more abundant, while those that impose fitness costs become less abundant (Thon *et al*., 2024). Despite the targeted metabolome showing diversity‐driven responses, our nontargeted analysis showed that features influenced by either SR, selection history, or environment history did not belong to a specific class but rather a mix of several classes of compounds. This finding reinforces the idea that changes in plant metabolomes are complex, resulting from interacting responses among metabolic features not confined to a single class of compounds. This underscores the need to be careful in interpreting compound classes always as functional classes when seeking explanations for plant defense responses. Additionally, it is important to consider that a plant is simultaneously exposed to (a)biotic factors, further supporting the concept of multivariate changes at the metabolomic level.

The interplay between phenotypic plasticity and adaptive changes at the metabolome level has been observed in previous studies. Research has highlighted that *P. lanceolata* demonstrates both plastic and adaptive capabilities in response to varying environmental conditions, particularly in its morphological and chemical traits (Bischoff *et al*., 2006; Skinner & Stewart, 2014; Medina‐van Berkum *et al*., 2024). However, the degree of these responses varies depending on the specific traits studied.

### Community history enhanced diversity‐driven responses at metabolic level

The impact of biodiversity on plant community performance increases with ecosystem ‘age’, as plant and soil processes change over time (Guerrero‐Ramírez *et al*., 2017; Meyer *et al*., 2017; Huang *et al*., 2018; Vogel *et al*., 2019). Given our findings that plants experienced selection pressures driven by plant diversity at the metabolome level, we further explored whether these effects were influenced by the history of the soil or plant community, based on the ΔBEF experiment (Vogel *et al*., 2019). Here, we provide evidence that plant diversity‐driven responses at the metabolic level are enhanced as the communities mature, promoting greater plasticity and adaptive responses to the increase of plant SR in the community.

Our findings indicate that the emission of VOCs from *P. lanceolata* showed stronger plasticity responses when plants shared history with the soil community, either only soil history or both soil and plant history (Fig. 5c). As a response to differences in plant diversity, the assembly of biotic communities and changes in soil nutrient availability over time create a history (Eisenhauer *et al*., 2024). Previous studies have shown that soil biota can significantly influence the emission and composition of volatiles in plants, involving both beneficial and detrimental ones (Fontana *et al*., 2009; Hammerbacher *et al*., 2019). Therefore, it is likely that the negative relationship between VOCs and SR strengthens over time, by the modulation of microbe‐mediated soil history relationships.

A previous study showed soil legacy effects in plant metabolome (Ristok *et al*., 2019). Although the overall metabolome composition was similar among the plants growing in different environments, we found that metabolic diversity in *selected* phytometers growing in their original environment had a stronger positive response to plant diversity compared with those in environments where they did not share community plant history (Fig. 5f). These results support the idea that the impact of biodiversity on plant performance increases with ecosystem age (Eisenhauer *et al*., 2024), not only at the level of morphological plant traits and plant performance but also at the chemical level.

### Conclusion

In summary, our study has shown a clear effect of plant diversity on *P. lanceolata* metabolome profiles, revealing contrasting responses between volatile and non‐volatile compound diversity. As SR in the surrounding environment increases, volatile diversity declines, whereas non‐volatile diversity takes the opposite trajectory, showing an increase. Moreover, our findings highlight the complex interplay between plasticity and adaptation in plant responses to their environment. While VOC emissions primarily show plasticity in response to species diversity in the surrounding community, non‐volatile compound production seems to involve both plastic and adaptive responses. Additionally, we demonstrated that plant and soil histories play critical roles in shaping plant metabolic responses to biodiversity over time. As soil and plant communities mature, these effects seem to intensify both the plastic and adaptive responses of plants to their surrounding community, emphasizing the dynamics of plant interactions at the metabolomic level. Further research is needed to disentangle the contributions of these mechanisms and to understand how they shape plant interactions within diverse ecological communities.

## Competing interests

None declared.

## Author contributions

PM‐B, FDG, WD, CR and SBU designed the research. PM‐B and FDG performed the field experiment. PM‐B and BR performed the chemical analysis. PM‐B analyzed the data and wrote the first draft of the manuscript. JG, CR, WD, FDG and SBU reviewed and edited the manuscript. All authors discussed the results, contributed substantially to the drafts, and gave final approval of the manuscript before the submission.

## Disclaimer

The New Phytologist Foundation remains neutral with regard to jurisdictional claims in maps and in any institutional affiliations.

## Supporting information


**Fig. S1** Layout of the long‐term grassland experiment ‘The Jena Experiment’ highlighting the plots used in this study.
**Fig. S2** Upset plot of the interactions of features whose intensity was significantly influenced by vegetation height, species richness, selection history, or their interaction.
**Fig. S3** Heatmap of leaf metabolic features in *Plantago lanceolata* significantly influenced by species richness and community history.
**Fig. S4** Upset plot of the interactions of features whose intensity was significantly influenced by vegetation height, species richness, community history, or their interaction.
**Table S1** Selection Experiment: Wald chi‐squared analysis of variance results for the linear mixed models of *naïve* and *selected Plantago lanceolata* phytometers across a diversity gradient based on leaf traits and leaf damage.
**Table S2** Community History Experiment: Wald chi‐squared analysis of variance results for the linear mixed models of *selected Plantago lanceolata* phytometers across a diversity gradient in different community history environments based on leaf traits and leaf damage.
**Table S3** List of volatile organic compounds identified in phytometers of *Plantago lanceolata* transplanted in the Jena Experiment.
**Table S4** Selection Experiment: Wald chi‐squared analysis of variance results for the linear mixed models of *naïve* and *selected Plantago lanceolata* phytometers across a diversity gradient based on volatile organic compound profiles.
**Table S5** Selection Experiment: Wald chi‐squared analysis of variance results for the linear mixed models of *naïve* and *selected Plantago lanceolata* phytometers across a diversity gradient based on untargeted metabolome diversity.
**Table S6** Selection Experiment: Wald chi‐squared analysis of variance results for the linear mixed models of *naïve* and *selected Plantago lanceolata* phytometers across a diversity gradient based on targeted defense metabolites.
**Table S7** Community History Experiment: Wald chi‐squared analysis of variance results for the linear mixed models of *selected Plantago lanceolata* phytometers across a diversity gradient in different community history environments based on volatile organic compound profiles.
**Table S8** Community History Experiment: Wald chi‐squared analysis of variance results for the linear mixed models of *selected Plantago lanceolata* phytometers across a diversity gradient in different community history environments based on untargeted metabolome diversity.
**Table S9** Community History Experiment: Wald chi‐squared analysis of variance results for the linear mixed models of *selected Plantago lanceolata* phytometers across a diversity gradient in different community history environments based on targeted defense compounds.
Please note: Wiley is not responsible for the content or functionality of any Supporting Information supplied by the authors. Any queries (other than missing material) should be directed to the *New Phytologist* Central Office.

## Data Availability

The R markdown notebook with the codes used (10.25829/S78S-EK50), leaf traits (10.25829/3YSY-WS93), leaf damage (10.25829/00KY-5246), volatile organic compounds (10.25829/DR77-W859), defense compounds based on targeted analysis (10.25829/0BJB-4549) and processed metabolome data (10.25829/JFN3-NY30, 10.25829/70B7-8675, 10.25829/Y3GP-7T72) are available through the Jena Experiment database (https://jexis.idiv.de). Raw metabolome data are available from MetaboLights (Yurekten *et al*., 2023; www.ebi.ac.uk/metabolights/MTBLS11792; Study Identifier: MTBLS11792).
