# Supplementary material for: Selection strengthens the relationship between plant diversity and the metabolic profile of Plantago lanceolata
Source: New Phytol. 2025 Jul 24;247(6):2982–97. doi: 10.1111/nph.70340 (PMC12371175; doi:10.1111/nph.70340)
Supplement: Supplementary file 1 — Fig. S1 Layout of the long‐term grassland experiment ‘The Jena Experiment’ highlighting the plots used in this study. Fig. S2 Upset plot of the interactions of features whose intensity was significantly influenced by vegetation height, species richness, selection history, or their interaction. Fig. S3 Heatmap of leaf metabolic features in Plantago lanceolata significantly influenced by species richness and community history. Fig. S4 Upset plot of the interactions of features whose intensity was significantly influenced by vegetation height, species richness, community history, or their interaction. Table S1 Selection Experiment: Wald chi‐squared analysis of variance results for the linear mixed models of naïve and selected Plantago lanceolata phytometers across a diversity gradient based on leaf traits and leaf damage. Table S2 Community History Experiment: Wald chi‐squared analysis of variance results for the linear mixed models of selected Plantago lanceolata phytometers across a diversity gradient in different community history environments based on leaf traits and leaf damage. Table S3 List of volatile organic compounds identified in phytometers of Plantago lanceolata transplanted in the Jena Experiment. Table S4 Selection Experiment: Wald chi‐squared analysis of variance results for the linear mixed models of naïve and selected Plantago lanceolata phytometers across a diversity gradient based on volatile organic compound profiles. Table S5 Selection Experiment: Wald chi‐squared analysis of variance results for the linear mixed models of naïve and selected Plantago lanceolata phytometers across a diversity gradient based on untargeted metabolome diversity. Table S6 Selection Experiment: Wald chi‐squared analysis of variance results for the linear mixed models of naïve and selected Plantago lanceolata phytometers across a diversity gradient based on targeted defense metabolites. Table S7 Community History Experiment: Wald chi‐squared analysis of variance results [file NPH-247-2982-s001.pdf]

New Phytologist

***New Phytologist* Supporting Information**

Article title: **Selection strengthens the relationship between plant diversity and the metabolic profile of *Plantago lanceolata***

Authors: Pamela Medina-van Berkum, Francesca De Giorgi, Beate Rothe, Walter Durka, Jonathan Gershenzon, Christiane Roscher, Sybille B. Unsicker

Article acceptance date: 2-June-2025

The following Supporting Information is available for this article:

Figure 1 Layout of the long-term grassland Experiment the Jena Experiment highlighting the plots used in this study. 3

Figure 2. Upset plot of the interactions of features whose intensity were significantly influenced by vegetation height, species richness, presence of legumes, selection history or their interactions. 4

Figure 3. Heatmap of leaf metabolic features in selected *Plantago lanceolata* significantly influenced by species richness and community history**.** 5

Figure 4 Upset plot of the interactions of features whose intensity were significantly influenced by vegetation height, species richness, community history or their interaction. 6

Table S1. **Selection Experiment:** Wald-chi-squared analysis of variance (ANOVA) results for the linear mixed models of *naïve* and *selected Plantago lanceolata* phytometers across a diversity gradient based on leaf traits and leaf damage. *7*

Table S2 **Community History Experiment:** Wald-chi-squared analysis of variance (ANOVA) results for the linear mixed models of *selected Plantago lanceolata* phytometers across a diversity gradient in different community history environments based on leaf traits and leaf damage. *9*

Table S3. List of volatile organic compounds (VOC) identified in phytometers of *Plantago lanceolata* transplanted in the Jena Experiment. *11*

Table S4. **Selection Experiment:** Wald-chi-squared analysis of variance (ANOVA) results for the linear mixed models of *naïve* and *selected Plantago lanceolata* phytometers across a diversity gradient based on volatile organic compound profiles. *12*

Table S5 **Selection Experiment:** Wald-chi-squared analysis of variance (ANOVA) results for the linear mixed models of *naïve* and *selected Plantago lanceolata* phytometers across a diversity gradient based on non-volatile untargeted metabolome diversity. *15*

Table S6 **Selection Experiment:** Wald-chi-squared analysis of variance (ANOVA) results for the linear mixed models of *naïve* and *selected Plantago lanceolata* phytometers across a diversity gradient based on targeted defense metabolites.______ *16*

Table S7 **Community History Experiment:** Wald-chi-squared analysis of variance (ANOVA) results for the linear mixed models of *selected Plantago lanceolata* phytometers across a diversity gradient in different community history environments based on volatile organic compounds profiles.______ *19*

Table S8. **Community History Experiment:** Wald-chi-squared analysis of variance (ANOVA) results for the linear mixed models of *selected Plantago lanceolata* phytometers across a diversity gradient in different community history environments based on untargeted metabolome diversity. ______ *22*

Table S9. **Community History Experiment:** Wald-chi-squared analysis of variance (ANOVA) results for the linear mixed models of *selected Plantago lanceolata* phytometers across a diversity gradient in different community history environments based on targeted defense compounds. ______ *23*

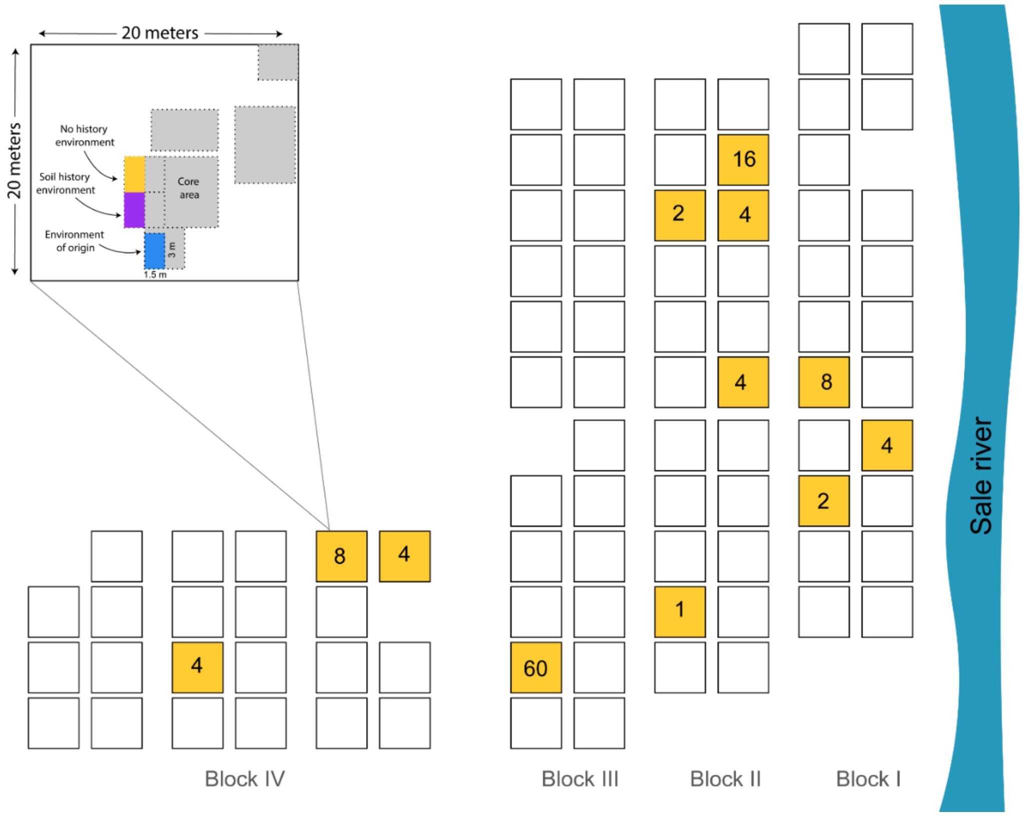

Figure 1 Layout of the long-term grassland experiment "The Jena Experiment" highlighting the plots used in this study. The Jena Experiment consists of 82 experimental plots (20 m × 20 m, 400 m^2 each) arranged along a gradient of plant species richness, ranging from monocultures to 60-species mixtures (1, 2, 4, 8, 16, and 60 species). It also includes a gradient of functional group richness (1–4 functional groups: grasses, small herbs, tall herbs, and legumes), composed of native species typical of Central European mesophilic grasslands. The experiment set up in four blocks in four blocks containing an equal number of plots, to account for natural variation in soil characteristics, the experiment was set up in four blocks containing an equal number of plots. All plots are regularly maintained by mowing and weeding twice per year (see Roscher et al., 2004, for more details). In this study, we selected the plots were *Plantago lanceolata* belonged to the sown species combinations (in yellow), covering a gradient in species richness from a *P. lanceolata* monoculture to a 60 plant species-mixture. Phytometers were transplanted into the ΔBEF (Biodiversity-Ecosystem Functioning) treatments corresponding to their original community. The ΔBEF experiment was established in 2016 and includes three subplots (1.5 m × 3 m) within each main plot, each representing different levels of community history. (1) Soil and plant history: 17-year-old plant communities, long-term control (from where the seeds were collected, their environment of origin). (2) Soil history: experimental environment in which plant species were removed while keeping the soil and resowing plot-specific plant species. (3) No history: experimental environment in which soil and plant layer were removed and replaced with arable field soil resown with plot-specific plant species (see Vogel et al., 2019 for more details).

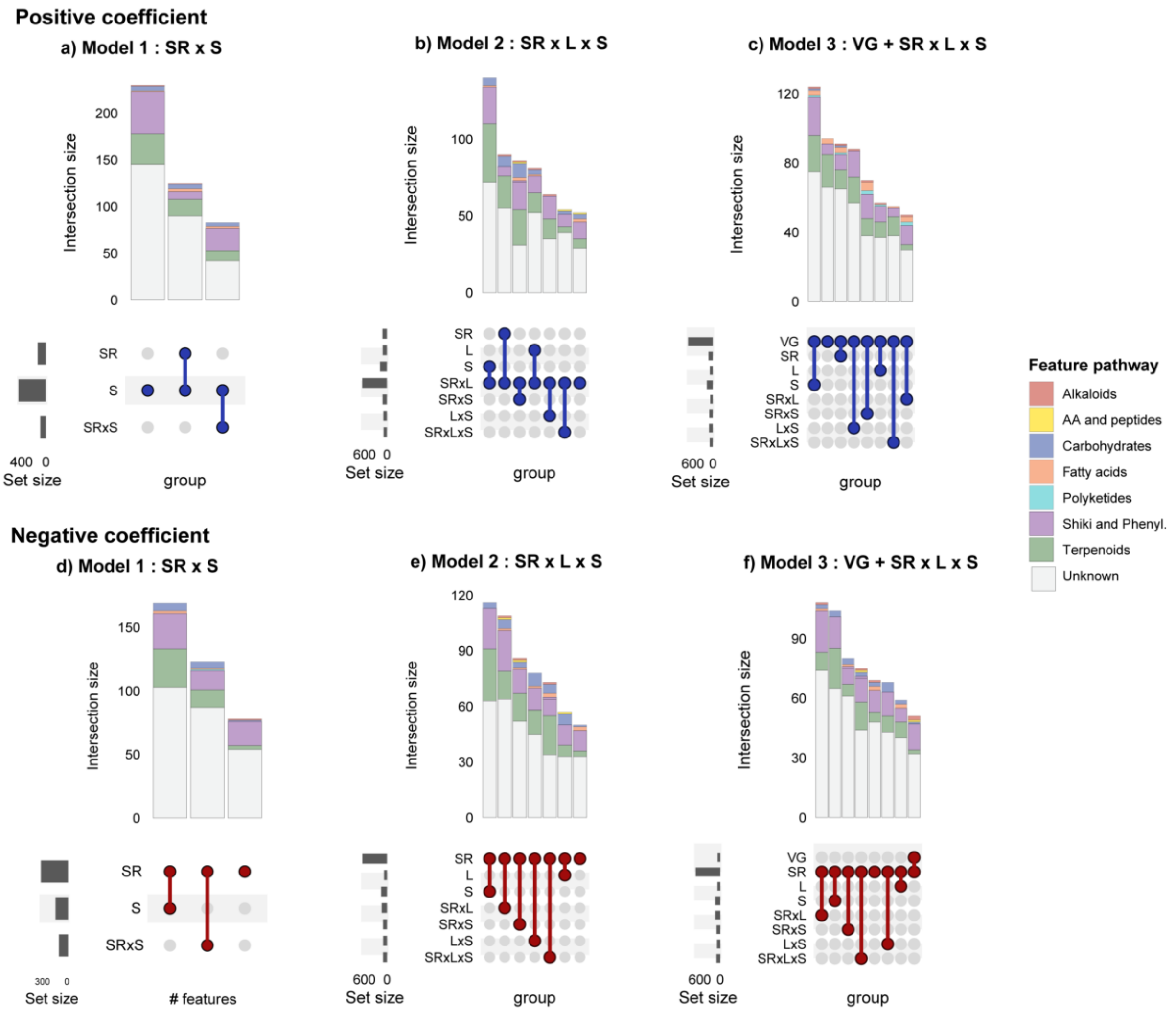

Figure 2. Upset plot of the interactions of features whose intensity were significantly influenced by vegetation height, species richness, presence of legumes, selection history or their interactions. Features positive correlated with the fixed factor (top section) a) Model 1: species richness x selection; b) Model 2: species richness x legumes x selection; c) Model 3: vegetation height x species richness x legumes x selection. Features negative correlated with the fixed factor (bottom section) a) Model 1: species richness x selection; b) Model 2: species richness x legumes x selection; c) Model 3: vegetation height x species richness x legumes x selection. The blue or read bars (bottom left) indicate the number of features whose intensity was significantly influenced by each fixed factor. The blue dots (connected with black lines) represent the intersections of features by each factor, and the bars (top) indicate the frequency of these intersections. Features were putatively classified by biosynthetic pathway. Effect of the treatments to the intensity was tested with generalized linear mixed regression model.

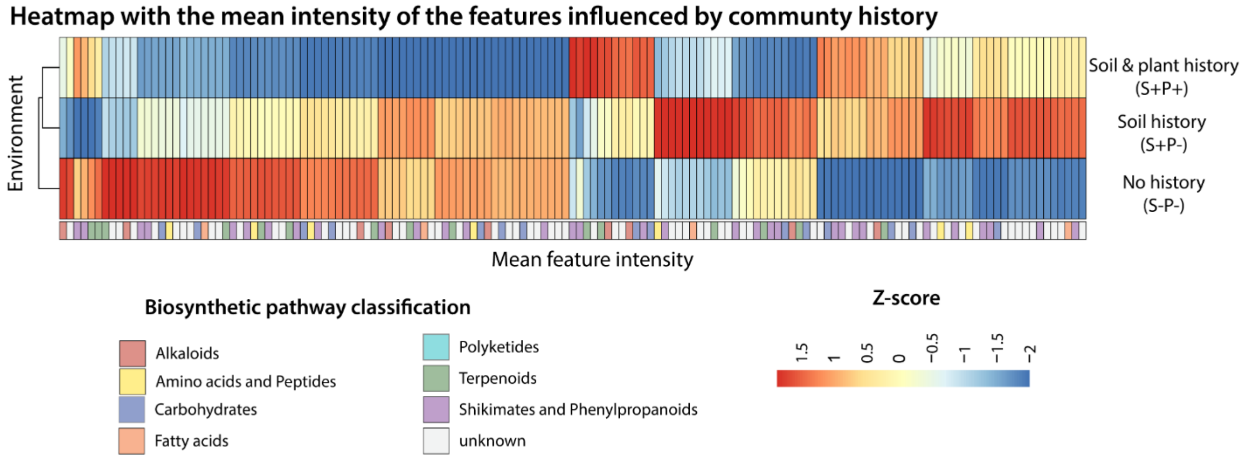

Figure 3. Heatmap of leaf metabolic features in selected Plantago lanceolata significantly influenced by species richness and community history. Effect of the treatments to the intensity was tested with generalized linear mixed regression model. The community history experiment compared the metabolomic profiles of selected plants grown in different environment treatments based on the δBEF experiment established in 2016(Vogel et al., 2019). No history (NH: –SH-PH): soil and plant layer removed, Soil history (SH: +SH-PH): only plant layer removed. In both treatments, new plot-specific plants species-mixtures were sowed. Soil-Plant history (SPH: +SH+PH): as control, same as core area stablished in 2002.

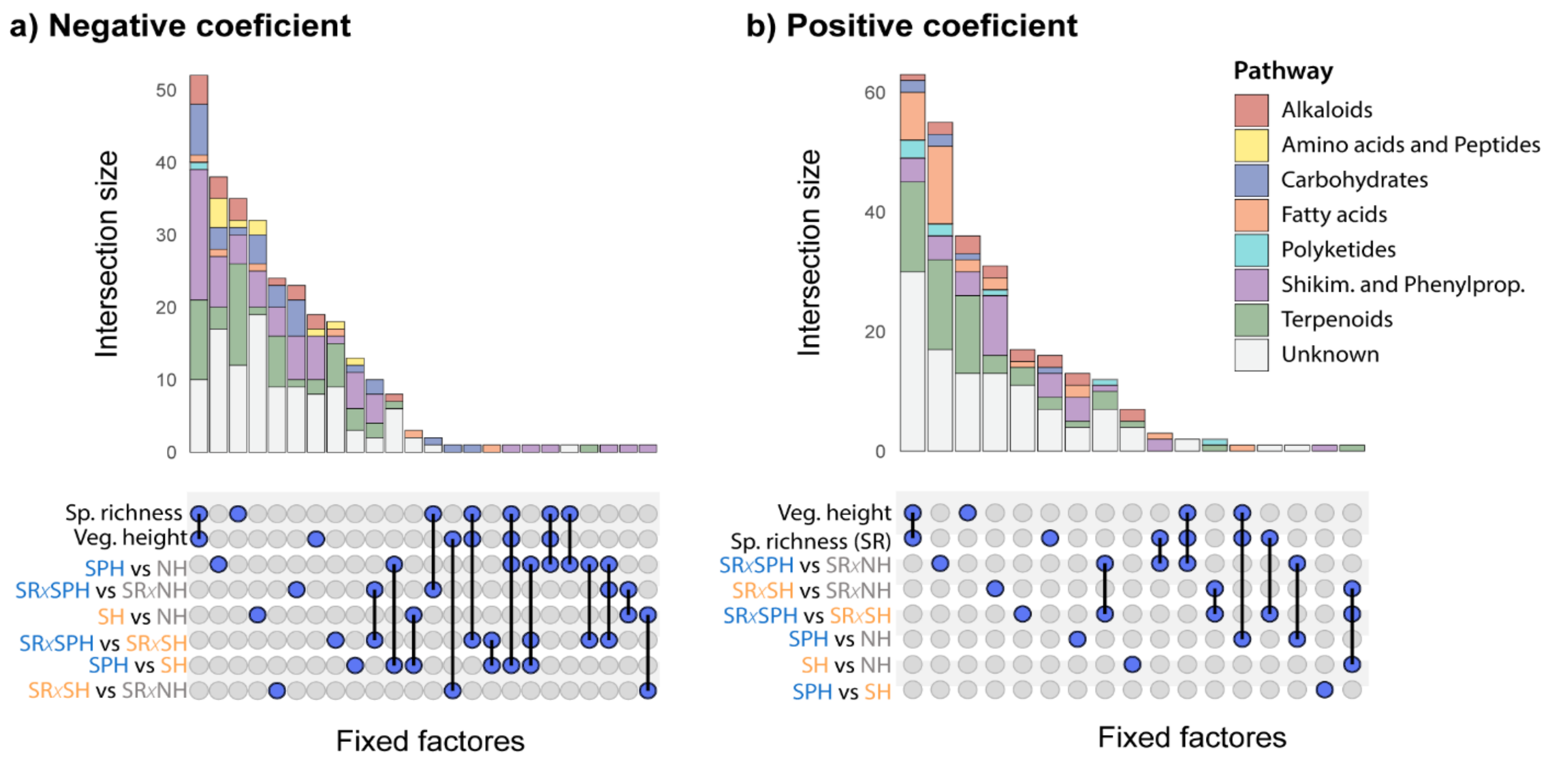

Figure 4 Upset plot of the interactions of features whose intensity were significantly influenced by vegetation height, species richness, community history or their interaction. a) Features that were negatively correlated with the treatment; b) Features that were positively correlated with the treatment. The blue bars (bottom left) indicate the number of metabolites whose intensity was significantly influenced by each fixed factor. The blue dots (connected with black lines) represent the intersections of metabolites by each factor, and the bars (top) indicate the frequency of these intersections. Features were putatively classified by biosynthetic pathway. Effect of the treatments to the feature intensity was tested with linear mixed regression model (intensity~ vegetation height + species richness * environment+ [1| block/plot]). The community history experiment compared the metabolomic profiles of selected plants grown in different environment treatments based on the δBEF experiment established in 2016 (Vogel et al., 2019). No history (NH: –SH-PH): soil and plant layer removed, Soil history (SH: +SH-PH): only plant layer removed. In both treatments, new plot-specific plants species-mixtures were sowed. Soil-Plant history (SPH: +SH+PH): as control, same as core area stablished in 2002.

Table S1. **Selection *Experiment:* Wald-chi-squared analysis of variance (ANOVA) results for the linear mixed models of *naïve* and *selected* *Plantago lanceolata* phytometers across a diversity gradient based on leaf traits and leaf damage.** The effects of vegetation height, plant species richness, selection history (*naïve* or *selected*) and legumes (presence or absence) on leaf traits and leaf damage were tested using mixed-effects models. Six models were run to disentangle the confounding effects: Model 1 examined species richness, selection treatment, and their interaction. Models 2 and 3 assessed legumes presence, either before or after species richness. Models 4-6 tested vegetation height, including it as a covariate. All models used plot nested within block as random effects. The table shows Chi-square (X^2) and p-values for fixed effects, with significant effects in bold ($P< 0.05$) and tendencies within brackets ($P < 0.1$). Data were transformed as needed to meet assumptions.

Trait	Explanatory variable	Model 1		Model 2		Model 3		Model 4		Model 5		Model 6		
		y~ SR S+ (1| block/ plot)*		*y~ SR*L* S+ (1| block/ plot)*		*y~ L*SR*S+ (1| block/ plot)*		*y~ VG+SR*S+ (1| block/ plot)*		*y~ VG+SR*L*S+ (1| block/ plot)*		*y~ VG+L*SR* S+ (1| block/ plot)*		
		X^2	***P***	***X^2***	***P***	***X^2***	***P***	***X^2***	***P***	***X^2***	***P***	***X^2***	***P***	
**Morphological leaf traits**														
Shoot biomass g DW glmer N= 169	Veg. height (VG)	NA	NA	NA	NA	NA	NA	**5.22**	**0.022**	**5.22**	**0.022**	**5.22**	**0.022**	Shoot biomass increased with increasing species richness only in *selected* phytometers.
	Species richness (SR)	1.48	0.224	1.48	0.224	5.24	**0.022**	0.06	0.810	0.06	0.810	[2.99]	*0.084*	
	Legume (L)	NA	NA	**7.23**	**0.007**	[3.47]	*0.062*	NA	NA	[3.47]	*0.063*	0.54	0.464	
	Selection (S)	0.00	0.969	0.02	0.896	0.02	0.896	0.02	0.892	0.02	0.895	0.02	0.895	
	SR x L	NA	NA	0.00	1.000	0.00	1.000	NA	NA	1.00	0.318	1.00	0.318	
	SR x S	**4.95**	**0.026**	**5.36**	**0.021**	[3.52]	*0.061*	**5.11**	**0.024**	**5.30**	**0.021**	2.72	*0.099*	
	L x S	NA	NA	**4.60**	**0.032**	**6.45**	**0.011**	NA	NA	[3.16]	*0.076*	**5.74**	**0.017**	
	SR x L x S	NA	NA	0.01	0.919	0.01	0.919	NA	NA	0.02	0.885	0.01	0.907	
	$R^2_{marginal/conditional}$	0.04/0.38		0.19/0.44		0.19/0.44		0.1/0.35		0.16/0.39		0.16/0.40		
Leaf length cm glmer N= 169	Veg. height (VG)	NA	NA	NA	NA	NA	NA	**29.02**	**0.000**	**29.02**	**<0.001**	**29.02**	**<0.001**	Increased with increasing vegetation height. Higher in plots with legumes.
	Species richness (SR)	[2.98]	*0.084*	[2.98]	*0.084*	2.12	0.146	2.03	0.154	2.03	0.154	0.03	0.864	
	Legume (L)	NA	NA	2.68	0.102	[3.54]	*0.060*	NA	NA	**6.45**	**0.011**	**8.45**	**0.004**	
	Selection (S)	0.03	0.859	0.03	0.856	0.03	0.855	0.01	0.920	0.01	0.918	0.01	0.918	
	SR x L	NA	NA	0.18	0.671	0.18	0.671	NA	NA	0.00	0.994	0.00	0.994	
	SR x S	1.90	0.168	1.82	0.178	2.39	0.122	2.45	0.117	1.93	0.165	2.41	0.121	
	L x S	NA	NA	**4.96**	**0.026**	**4.39**	**0.036**	NA	NA	[3.86]	*0.050*	3.38	*0.066*	
	SR x L x S	NA	NA	1.18	0.278	1.19	0.275	NA	NA	0.79	0.375	0.79	0.375	
	$R^2_{marginal/conditional}$	0.01/0.10		0.01/0.09		0.01/0.09		0.01/0.09		0.02/0.10		0.02/0.10		
Leaf greenness SPAD glmer	Veg. height (VG)	NA	NA	NA	NA	NA	NA	0.17	0.684	0.17	0.684	0.17	0.684	Plots with legumes had higher SPAD.
	Species richness (SR)	0.64	0.423	0.64	0.423	[3.44]	*0.064*	1.42	0.234	1.42	0.234	1.36	0.243	
	Legume (L)	NA	NA	**9.01**	**0.003**	**6.21**	**0.013**	NA	NA	**9.11**	**0.003**	**9.16**	**0.002**	
	Selection (S)	0.90	0.344	0.93	0.335	0.93	0.335	0.95	0.329	0.86	0.353	0.86	0.353	
	SR x L	NA	NA	0.94	0.333	0.94	0.333	NA	NA	1.57	0.210	1.57	0.210	
	SR x S	0.07	0.793	0.11	0.738	0.06	0.808	0.07	0.792	0.17	0.684	0.10	0.751	
	L x S	NA	NA	0.33	0.563	0.39	0.533	NA	NA	0.29	0.588	0.36	0.549	
	SR x L x S	NA	NA	[3.30]	*0.069*	[3.30]	*0.069*	NA	NA	[3.04]	*0.081*	[3.04]	*0.081*	
	$R^2_{marginal/conditional}$	0.02/NA		0.11/NA		0.11/NA		0.03/NA		0.12/NA		0.12/NA		
Flowering status	Veg. height (VG)	NA	NA	NA	NA	NA	NA	2.59	0.107	2.59	0.107	2.59	0.107	
	Species richness (SR)	2.66	0.103	2.66	0.103	1.89	0.169	0.97	0.324	0.97	0.324	1.16	0.282	
	Legume (L)	NA	NA	1.73	0.189	2.49	0.114	NA	NA	0.93	0.336	0.74	0.389	

(yes/no) binomial	Selection (S)	0.03	0.864	0.02	0.890	0.02	0.890	0.02	0.876	0.02	0.891	0.02	0.891	
	SR x L	NA	NA	1.32	0.251	1.32	0.251	NA	NA	1.22	0.270	1.22	0.270	
	SR x S	[3.73]	*0.053*	[3.69]	*0.055*	[3.55]	*0.060*	[3.74]	*0.053*	[3.69]	*0.055*	[3.55]	*0.060*	
	L x S	NA	NA	0.04	0.844	0.18	0.674	NA	NA	0.04	0.846	0.18	0.675	
	SR x L x S	NA	NA	0.33	0.566	0.33	0.566	NA	NA	0.33	0.566	0.33	0.566	
	R^2 marginal/conditional	0.27/NA		0.23/0.53		0.23/0.53		0.29/NA		0.23/0.53		0.23/0.53		
**Percentage of leaf damage**														
Herbivore damage % arcsin N= 56	Veg. height (VG)	NA	NA	NA	NA	NA	NA	0.76	0.383	0.76	0.383	0.76	0.383	
	Species richness (SR)	1.10	0.294	1.10	0.294	1.05	0.305	0.38	0.537	0.38	0.537	0.13	0.714	
	Legume (L)	NA	NA	0.05	0.821	0.10	0.752	NA	NA	0.16	0.691	0.41	0.524	
	Selection (S)	0.28	0.594	0.26	0.610	0.26	0.610	0.30	0.583	0.26	0.609	0.26	0.609	
	SR x L	NA	NA	0.64	0.422	0.64	0.422	NA	NA	1.11	0.293	1.11	0.293	
	SR x S	0.04	0.835	0.09	0.760	0.02	0.886	0.04	0.839	0.14	0.711	0.04	0.840	
	L x S	NA	NA	0.81	0.368	0.88	0.348	NA	NA	0.88	0.348	0.98	0.323	
	SR x L x S	NA	NA	0.14	0.710	0.14	0.710	NA	NA	0.10	0.757	0.10	0.757	
	R^2 marginal/conditional	0.04/NA		0.09/NA		0.09/NA		0.04/NA		0.12/NA		0.12/NA		
Pathogen damage % arcsin N= 56	Veg. height (VG)	NA	NA	NA	NA	NA	NA	**15.22**	**<0.001**	**15.22**	**<0.001**	**15.22**	**<0.001**	Decrease with increasing vegetation height
	Species richness (SR)	1.52	0.218	1.52	0.218	0.74	0.390	0.78	0.376	0.78	0.376	0.93	0.334	
	Legume (L)	NA	NA	[3.00]	*0.083*	[3.78]	*0.052*	NA	NA	0.15	0.698	0.00	0.986	
	Selection (S)	1.56	0.211	1.30	0.253	1.30	0.253	1.21	0.271	1.27	0.260	1.27	0.260	
	SR x L	NA	NA	1.16	0.282	1.16	0.282	NA	NA	0.19	0.663	0.19	0.663	
	SR x S	0.01	0.934	0.05	0.815	0.13	0.721	0.09	0.770	0.05	0.826	0.10	0.749	
	L x S	NA	NA	0.64	0.424	0.57	0.452	NA	NA	0.35	0.555	0.29	0.588	
	SR x L x S	NA	NA	2.02	0.155	2.02	0.155	NA	NA	1.43	0.232	1.43	0.232	
	R^2 marginal/conditional	0.14/0.71		0.36/0.73		0.36/0.73		0.59/0.69		0.61/0.72		0.61/0.72		
Total leaf damage % arcsin N= 56	Veg. height (VG)	NA	NA	NA	NA	NA	NA	**14.83**	**<0.001**	**14.83**	**<0.001**	**14.83**	**<0.001**	Decrease with increasing vegetation height
	Species richness (SR)	1.16	0.281	1.16	0.281	0.46	0.500	2.26	0.133	2.26	0.133	1.96	0.162	
	Legume (L)	NA	NA	**4.05**	**0.044**	**4.76**	**0.029**	NA	NA	0.00	0.995	0.30	0.583	
	Selection (S)	[2.77]	[0.096]	2.25	0.133	2.25	0.133	2.19	0.139	2.19	0.139	2.19	0.139	
	SR x L	NA	NA	1.47	0.225	1.47	0.225	NA	NA	0.00	0.956	0.00	0.956	
	SR x S	0.03	0.865	0.14	0.707	0.20	0.654	0.16	0.686	0.16	0.690	0.20	0.658	
	L x S	NA	NA	0.25	0.614	0.19	0.659	NA	NA	0.09	0.767	0.05	0.823	
	SR x L x S	NA	NA	1.21	0.272	1.21	0.272	NA	NA	0.74	0.389	0.74	0.389	
	R^2 marginal/conditional	0.12/0.67		0.56/NA		0.56/NA		0.59/0.65		0.6/0.67		0.6/0.67		

Table S2 Community History Experiment: Wald-chi-squared analysis of variance (ANOVA) results for the linear mixed models of *selected Plantago lanceolata* phytometers across a diversity gradient in different community history environments based on leaf traits and leaf damage. The effects of vegetation height, species richness, experimental environment (S+P+, S+P-, S-P-) and legumes (presence or absence) on leaf traits and leaf damage were tested using mixed-effects models. The community history experiment compared the targeted defense compounds of selected phytometers grown in different experimental environments based on the ΔBEF experiment established in 2016 (Vogel et al., 2019). Six models were run to disentangle the confounding effects: Model 1 examined species richness, environment treatment, and their interaction. Models 2 and 3 assessed legumes presence, either before or after species richness. Models 4-6 tested vegetation height, including it as a covariate. All models used plot nested within block as random effects. The table shows N, R^2, Chi-square (X^2) and p-values for fixed effects, with significant effects in bold ($P< 0.05$) and tendencies within brackets ($P < 0.1$). Data were transformed as needed to meet assumptions.

Leaf trait	Explanatory variable	**Model 1** *y~ SR* E+ (1| block/ plot)*		**Model 2** *y~ SR*L* E+ (1| block/ plot)*		**Model 3** *y~ L*SR*E+ (1| block/ plot)*		**Model 4** *y~ VG+SR*E+ (1| block/ plot)*		**Model 5** *y~ VG+SR*L* E+ (1| block/ plot)*		**Model 6** *y~ VG+L*SR* E+ (1| block/ plot)*		Pattern
		X²	***P***	***X²***	***P***	***X²***	***P***	***X²***	***P***	***X²***	***P***	***X²***	***P***	
**Morphological leaf traits**														
Shoot biomass g DW glmer N= 169	Veg. height (VG)	NA	NA	NA	NA	NA	NA	0.30	0.586	0.30	0.586	0.30	0.586	Shoot biomass increased with increasing species richness in *selected* phytometers only when they grew in their environment of origin. Effects are stronger in plots without legumes
	Species richness (SR)	1.03	0.309	1.03	0.309	1.01	0.314	0.76	0.384	0.76	0.384	1.06	0.303	
	Legume (L)	NA	NA	2.25	0.133	2.27	0.13	NA	NA	2.31	0.128	2.01	0.156	
	Environment (E)	1.61	0.456	1.55	0.461	1.55	0.461	1.59	0.451	1.66	0.436	1.66	0.436	
	SR x L	NA	NA	0.23	0.610	0.23	0.610	NA	NA	0.29	0.592	0.29	0.592	
	SR x E	**9.86**	**0.007**	**9.94**	**0.007**	**8.20**	**0.016**	**10.10**	**0.006**	**9.70**	**0.007**	**7.82**	**0.020**	
	L x E	NA	NA	0.94	0.62	2.69	0.260	NA	NA	0.94	0.617	2.83	0.242	
	SR x L x E	NA	NA	**15.52**	**< 0.001**	**15.52**	**< 0.001**	NA	NA	**15.51**	**< 0.001**	**15.51**	**< 0.001**	
	R^2 marginal/conditional	0.05/0.05		0.13/0.39		0.13/0.38		0.06/0.06		0.4/0.83		0.42/0.84		
Leaf length cm glmer N= 169	Veg. height (VG)	NA	NA	NA	NA	NA	NA	0.01	0.905	0.01	0.905	0.01	0.905	Increased with increasing species richness regardless of the environment treatment
	Species richness (SR)	**4.71**	**0.030**	**4.71**	**0.030**	**4.88**	**0.027**	**5.16**	**0.023**	**5.16**	**0.023**	**5.77**	**0.016**	
	Legume (L)	NA	NA	2.27	0.132	2.10	0.147	NA	NA	[2.82]	*0.093*	2.20	0.138	
	Environment (E)	[5.31]	*0.070*	[4.98]	*0.083*	[4.98]	*0.083*	[5.2]	*0.074*	[4.85]	*0.089*	[4.85]	*0.089*	
	SR x L	NA	NA	0.02	0.897	0.02	0.897	NA	NA	0.05	0.818	0.05	0.818	
	SR x E	0.73	0.695	0.72	0.699	0.50	0.777	0.59	0.745	0.50	0.779	0.33	0.850	
	L x E	NA	NA	[4.66]	*0.097*	[4.87]	*0.088*	NA	NA	[5.27]	*0.072*	[5.45]	*0.066*	
	SR x L x E	NA	NA	3.98	0.137	3.98	0.137	NA	NA	3.50	0.174	3.50	0.174	
	R^2 marginal/conditional	0.1/0.21		0.14/0.23		0.14/0.23		0.1/0.22		0.14/0.24		0.14/0.24		
Leaf greenness SPAD glmer N= 169	Veg. height (VG)	NA	NA	NA	NA	NA	NA	**7.56**	**0.006**	**7.56**	**0.006**	**7.56**	**0.006**	Plots with legumes had higher SPAD, while SPAD decreased with
	Species richness (SR)	2.11	0.1463	2.11	0.1463	**13.99**	**< 0.001**	[3.65]	*0.056*	[3.65]	*0.056*	**2.13**	**0.144**	
	Legume (L)	NA	NA	**6.53**	**0.011**	2.53	0.112	NA	NA	**13.68**	**< 0.001**	**15.19**	**< 0.001**	
	Environment (E)	0.42	0.810	0.29	0.863	0.29	0.863	0.14	0.932	0.46	0.793	0.46	0.793	

	SR x L	NA	NA	1.90	0.168	1.90	0.168	NA	NA	2.37	0.124	2.37	0.124	increasing vegetation height in their surroundings
	SR x E	2-95	0.229	2.06	0.357	1.23	0.540	1.52	0.468	1.35	0.510	0.64	0.727	
	L x E	NA	NA	2.03	0.362	2.86	0.239	NA	NA	2.70	0.260	3.40	0.182	
	SR x L x E	NA	NA	2.17	0.338	2.17	0.338	NA	NA	3.39	0.183	3.39	0.183	
	R^2 $_{marginal/conditional}$	0.05/0.05		0.12/0.12		0.12/0.12		0.12/0.12		0.13/0.18		0.13/0.18		
Flowering status (yes/no) binomial/glmer N= 169	Veg. height (VG)	NA	NA	NA	NA	NA	NA	0.11	0.735	0.11	0.735	0.11	0.735	Shoot biomass increased with increasing species richness in *selected* phytometers only when they grew in their environment of origin. Effects are stronger in plots without legumes
	Species richness (SR)	0.38	0.539	0.38	0.539	0.18	0.674	0.81	0.368	0.81	0.368	1.12	0.289	
	Legume (L)	NA	NA	[2.87]	*0.090*	[3.07]	*0.080*	NA	NA	**4.12**	**0.042**	[3.81]	*0.051*	
	Environment (E)	2.46	0.292	2.44	0.295	2.44	0.295	2.03	0.362	1.65	0.438	1.65	0.438	
	SR x L	NA	NA	0.05	0.822	0.05	0.822	NA	NA	0.12	0.724	0.12	0.724	
	SR x E	**18.12**	**< 0.001**	**18.65**	**< 0.001**	**15.40**	**< 0.001**	18.00	**< 0.001**	18.31	**< 0.001**	**15.14**	**0.001**	
	L x E	NA	NA	3.60	0.166	**6.84**	**0.033**	NA	NA	4.17	0.124	**7.35**	**0.025**	
	SR x L x E	NA	NA	[4.76]	*0.092*	[4.76]	*0.092*	NA	NA	[4.74]	*0.093*	[4.74]	*0.093*	
	R^2 $_{marginal/conditional}$	0.21/0.28		0.21/0.28		0.21/0.28		0.15/0.25		0.23/NA		0.23/NA		
**Percentage of leaf damage**														
Herbivore damage % arcsin N= 80	Veg. height (VG)	NA	NA	NA	NA	NA	NA	1.06	0.304	1.06	0.304	1.06	0.304	
	Species richness (SR)	2.08	0.149	2.08	0.149	1.78	0.183	1.04	0.308	1.04	0.308	1.26	0.262	
	Legume (L)	NA	NA	0.41	0.523	0.72	0.397	NA	NA	0.44	0.509	0.22	0.640	
	Environment (E)	1.23	0.539	1.45	0.483	1.45	0.483	1.46	0.483	1.44	0.488	1.44	0.488	
	SR x L	NA	NA	0.55	0.457	0.55	0.457	NA	NA	0.58	0.446	0.58	0.446	
	SR x E	0.89	0.641	1.01	0.603	0.96	0.618	0.93	0.627	1.03	0.597	1.05	0.591	
	L x E	NA	NA	2.22	0.330	2.26	0.323	NA	NA	2.49	0.288	2.47	0.291	
	SR x L x E	NA	NA	2.37	0.306	2.37	0.306	NA	NA	2.54	0.281	2.54	0.281	
	R^2 $_{marginal/conditional}$	0.06/0.06		0.13/0.13		0.13/0.13		0.06/0.07		0.13/NA		0.13/0.13		
Pathogen damage % arcsin N= 80	Veg. height (VG)	NA	NA	NA	NA	NA	NA	1.08	0.298	1.08	0.298	1.08	0.298	Decrease with increasing species richness only in phytometers that grew with soil history
	Species richness (SR)	0.49	0.482	0.49	0.482	0.09	0.760	0.01	0.925	0.01	0.925	0.01	0.934	
	Legume (L)	NA	NA	[3.56]	*0.059*	**3.96**	**0.047**	NA	NA	[3.05]	*0.081*	[3.05]	*0.081*	
	Environment (E)	1.79	0.408	1.71	0.425	1.71	0.425	2.09	0.351	1.82	0.403	1.82	0.403	
	SR x L	NA	NA	1.32	0.250	1.32	0.250	NA	NA	1.38	0.241	1.38	0.241	
	SR x E	**8.25**	**0.016**	**8.71**	**0.013**	**6.49**	**0.039**	**9.36**	**0.009**	**9.47**	**0.009**	**7.30**	**0.026**	
	L x E	NA	NA	1.25	0.536	3.47	0.176	NA	NA	1.23	0.541	3.40	0.183	
	SR x L x E	NA	NA	0.05	0.974	0.05	0.974	NA	NA	0.56	0.754	0.56	0.754	
	R^2 $_{marginal/conditional}$	0.1/0.45		0.35/0.35		0.35/0.35		0.19/0.35		0.35/0.35		0.35/0.35		
Total leaf damage % arcsin N= 80	Veg. height (VG)	NA	NA	NA	NA	NA	NA	**4.29**	**0.038**	**4.29**	**0.038**	**4.29**	**0.038**	Decreased with increasing vegetation height
	Species richness (SR)	0.51	0.474	0.51	0.474	0.03	0.863	0.01	0.931	0.01	0.931	0.04	0.849	
	Legume (L)	NA	NA	[2.77]	*0.096*	[3.25]	*0.072*	NA	NA	0.76	0.384	0.73	0.394	
	Environment (E)	2.97	0.226	2.76	0.252	2.76	0.252	2.77	0.251	2.63	0.268	2.63	0.268	
	SR x L	NA	NA	1.60	0.206	1.60	0.206	NA	NA	1.69	0.193	1.69	0.193	
	SR x E	**6.19**	**0.045**	**6.56**	**0.038**	4.24	0.120	**7.77**	**0.021**	**7.91**	**0.019**	[5.46]	*0.065*	
	L x E	NA	NA	2.23	0.327	4.56	0.102	NA	NA	1.43	0.488	3.88	0.144	
	SR x L x E	NA	NA	0.16	0.921	0.16	0.921	NA	NA	0.39	0.823	0.39	0.823	
	R^2 $_{marginal/conditional}$	0.1/0.41		0.32/0.32		0.32/0.32		0.27/0.27		0.33/0.33		0.33/0.33		

Table S3. List of volatile organic compounds (VOC) identified in phytometers of *Plantago lanceolata* transplanted in the Jena Experiment. Headspace VOC collection of *P. lanceolata* was performed one year after transplantation using a push-pull system for two hours. Individual plants were enclosed with PET and tied at the bottom using cable binders and sponge to avoid damage. Ambient air entered the system after passing through an activated charcoal filter at a flow rate of 7 mL/min and was pumped out through a trap a rate of 4 mL/min. The trap contained 25 mg Porapak Q adsorbent in a Teflon tube that was inserted in the PET bag. Compounds are sorted by chemical class and retention time (RT in min in the GC-FID and GC-MS).
† symbol represents compounds identified by comparison to authentic standards, otherwise, they were identified by comparison of mass spectra and retention times to those in Willey or Nist Mass library.

Compound	Class	RT GC-FID	RT GC-MS
pseudocumene	Aromatic	8.02	7.76
1-methyl-3-propylbenzene	Aromatic	10.18	9.89
3-ethylbenzaldehyde	Aromatic	12.77	13.05
4′-ethylacetophenone	Aromatic	15.37	15.51
2-hexanol	Fatty acid derivate	4.40	4.11
4-methyloctane	Fatty acid derivate	4.69	4.32
E-2-hexanal †	Fatty acid derivate (GLV)	5.23	5.05
Z-3-hexenol	Fatty acid derivate (GLV)	5.60	5.11
1-octen-3-ol †	Fatty acid derivate	8.57	8.10
3-octanone	Fatty acid derivate	8.82	8.27
3-octanol †	Fatty acid derivate	8.91	8.50
1-octen-3-yl acetate	Fatty acid derivate	11.73	11.46
E-3-hexen-1-ol acetate †	Fatty acid derivate (GLV)	9.15	8.79
hexyl acetate	Fatty acid derivate (GLV)	9.32	8.96
E-2-hexenyl acetate	Fatty acid derivate (GLV)	9.38	9.29
tricyclene	Monoterpene	7.04	6.66
α-pinene †	Monoterpene	7.30	6.88
sabinene †	Monoterpene	7.83	7.88
β-pinene †	Monoterpene	8.27	7.94
E-β-ocimene †	Monoterpene	10.08	9.84
β -ocimene †	Monoterpene	10.14	9.81
DMNT †	Homoterpene	11.78	11.55
α-copaene †	Sesquiterpene	17.76	17.70
β-elemene †	Sesquiterpene	18.19	18.05
β-caryophyllene †	Sesquiterpene	18.75	18.65
α-bergamotene	Sesquiterpene	19.04	18.96
germacreneD	Sesquiterpene	20.03	19.96
unknown sesquiterpene	Sesquiterpene	20.08	20.02
unknown 1	Other	9.23	8.88
unknown 2	Other	10.25	10.03
unknown 3	Other	10.47	10.26

Table S4. Selection Experiment: Wald-chi-squared analysis of variance (ANOVA) results for the linear mixed models of naïve and selected *Plantago lanceolata* phytometers across a diversity gradient based on volatile organic compound profiles. The effects of vegetation height, plant species richness, selection history (*naïve* or *selected*) and legumes (presence or absence) on untargeted metabolome diversity were tested using mixed-effects models. Six models were run to disentangle the confounding effects: Model 1 examined species richness, selection treatment, and their interaction. Models 2 and 3 assessed legumes presence, either before or after species richness. Models 4-6 tested vegetation height, including it as a covariate. All models used plot nested within block as random effects. The table shows Chi-square (X^2) and p-values for fixed effects, with significant effects in bold ($P< 0.05$) and tendencies within brackets ($P < 0.1$). Data were transformed as needed to meet assumptions.

Trait	Explanatory factor	**Model 1**		**Model 2**		**Model 3**		**Model 4**		**Model 5**		**Model 6**		Pattern
		y~ SR* S+ (1| block/ plot)		y~ SR*L* S+ (1| block/ plot)		y~ L*SR*S+ (1| block/ plot)		y~ VG+SR*S+ (1| block/ plot)		y~ VG+SR*L*S+ (1| block/ plot)		y~ VG+L*SR* S+ (1| block/ plot)		
		X²	***P***	***X²***	***P***	***X²***	***P***	***X²***	***P***	***X²***	***P***	***X²***	***P***	
**Emission of volatile organic compounds**														
Aromatic sqrt N= 60	Veg. height	NA	NA	NA	NA	NA	NA	**4.57**	**0.033**	**4.57**	**0.033**	**4.57**	**0.033**	Decrease with increasing vegetation height. Effect stronger in communities with legumes
	Species richness (SR)	**5.60**	**0.018**	**5.60**	**0.018**	**5.29**	**0.021**	1.03	0.310	1.03	0.310	0.69	0.407	
	Legume (L)	NA	NA	0.01	0.916	0.32	0.572	NA	NA	1.56	0.211	1.91	0.167	
	Selection (S)	0.12	0.734	0.12	0.731	0.12	0.731	0.12	0.734	0.31	0.578	0.31	0.578	
	SR x L	NA	NA	**6.74**	**0.009**	**6.74**	**0.009**	NA	NA	**5.00**	**0.025**	**5.00**	**0.025**	
	SR x S	1.72	0.190	2.29	0.130	1.97	0.160	1.72	0.190	2.31	0.129	2.00	0.157	
	L x S	NA	NA	0.39	0.533	0.71	0.400	NA	NA	0.40	0.528	0.70	0.402	
	SR x L x S	NA	NA	0.21	0.646	0.21	0.646	NA	NA	0.19	0.662	0.19	0.662	
	$R^2_{marginal/conditional}$	0.48/NA		0.35/NA		0.35/NA		0.48/NA		0.35/NA		0.35/NA		
Green leaf volatiles log10 N= 60	Veg. height	NA	NA	NA	NA	NA	NA	1.16	0.282	1.16	0.282	1.16	0.282	
	Species richness (SR)	0.39	0.533	0.39	0.533	0.13	0.718	0.02	0.893	0.02	0.893	0.13	0.718	
	Legume (L)	NA	NA	1.92	0.165	2.18	0.140	NA	NA	1.15	0.284	1.04	0.309	
	Selection (S)	0.01	0.919	0.01	0.927	0.01	0.927	0.02	0.901	0.01	0.930	0.01	0.930	
	SR x L	NA	NA	0.45	0.503	0.45	0.503	NA	NA	0.65	0.419	0.65	0.419	
	SR x S	0.49	0.485	0.70	0.402	0.50	0.481	0.52	0.473	0.76	0.383	0.55	0.460	
	L x S	NA	NA	0.51	0.474	0.72	0.397	NA	NA	0.50	0.480	0.71	0.399	
	SR x L x S	NA	NA	0.16	0.693	0.16	0.693	NA	NA	0.14	0.710	0.14	0.710	
	$R^2_{marginal/conditional}$	0.01/NA		0.07/NA		0.07/NA		0.03/NA		0.07/NA		0.07/NA		
Monoterpene sqrt N= 60	Veg. height	NA	NA	NA	NA	NA	NA	0.36	0.551	0.36	0.551	1.18	0.278	
	Species richness (SR)	1.70	0.193	1.70	0.193	1.65	0.199	[3.63]	*0.057*	[3.63]	*0.057*	1.77	0.184	
	Legume (L)	NA	NA	0.43	0.514	0.47	0.491	NA	NA	0.15	0.697	1.54	0.215	
	Selection (S)	0.01	0.940	0.00	0.963	0.00	0.963	0.01	0.939	0.01	0.921	0.16	0.687	
	SR x L	NA	NA	0.04	0.837	0.04	0.837	NA	NA	0.23	0.633	0.02	0.887	
	SR x S	1.37	0.242	1.25	0.264	1.23	0.268	1.32	0.251	1.55	0.214	1.50	0.221	
	L x S	NA	NA	0.00	0.972	0.02	0.886	NA	NA	0.00	0.966	0.08	0.775	
	SR x L x S	NA	NA	1.32	0.251	1.32	0.251	NA	NA	1.67	0.196	0.40	0.527	
	$R^2_{marginal/conditional}$	0.07/NA		0.1/NA		0.1/NA		0.13/NA		0.18/NA		0.14/NA		
	Veg. height	NA	NA	NA	NA	NA	NA	**6.21**	**0.013**	**6.21**	**0.013**	**6.21**	**0.013**	

Sesquiterpene<br>log10<br>N= 60	Species richness (SR)	0.02	0.884	0.02	0.884	1.18	0.277	**6.93**	**0.008**	**6.93**	**0.008**	**5.98**	**0.014**	Decrease with increasing species richness. Effect stronger in communities with legumes
	Legume (L)	NA	NA	**11.59**	**0.001**	**10.43**	**0.001**	NA	NA	**5.19**	**0.023**	**6.13**	**0.013**	
	Selection (S)	0.27	0.606	0.05	0.821	0.05	0.821	0.17	0.678	0.10	0.756	0.10	0.756	
	SR x L	NA	NA	0.80	0.371	0.80	0.371	NA	NA	0.11	0.743	0.11	0.743	
	SR x S	0.27	0.605	0.00	0.992	0.02	0.896	0.06	0.804	0.03	0.858	0.00	0.970	
	L x S	NA	NA	0.88	0.347	0.87	0.352	NA	NA	0.90	0.342	0.93	0.334	
	SR x L x S	NA	NA	0.63	0.429	0.63	0.429	NA	NA	0.47	0.493	0.47	0.493	
	R^2 marginal/conditional	0.01/0.42		0.36/NA		0.36/NA		0.4/NA		0.45/NA		0.45/NA		
Others<br>sqrt<br>N= 60	Veg. height	NA	NA	NA	NA	NA	NA	0.07	0.794	0.07	0.794	0.07	0.794	Decrease with increasing species richness.
	Species richness (SR)	**5.45**	**0.020**	**5.45**	**0.020**	**5.78**	**0.016**	**7.36**	**0.007**	**7.36**	**0.007**	**7.60**	**0.006**	
	Legume (L)	NA	NA	0.33	0.567	0.00	0.971	NA	NA	0.28	0.595	0.04	0.847	
	Selection (S)	0.15	0.696	0.15	0.701	0.15	0.701	0.15	0.695	0.16	0.686	0.16	0.686	
	SR x L	NA	NA	0.01	0.919	0.01	0.919	NA	NA	0.85	0.358	0.85	0.358	
	SR x S	0.09	0.770	0.08	0.774	0.18	0.670	0.06	0.802	0.13	0.718	0.25	0.615	
	L x S	NA	NA	0.75	0.386	0.65	0.420	NA	NA	0.72	0.396	0.60	0.439	
	SR x L x S	NA	NA	0.02	0.886	0.02	0.886	NA	NA	0.05	0.820	0.05	0.820	
	R^2 marginal/conditional	0.18/NA		0.2/NA		0.2/NA		0.22/NA		0.25/NA		0.25/NA		
Total emission<br>log10<br>N= 60	Veg. height	NA	NA	NA	NA	NA	NA	1.31	0.253	1.31	0.253	1.31	0.253	
	Species richness (SR)	[2.76]	*0.097*	[2.76]	*0.097*	2.14	0.143	1.63	0.202	1.63	0.202	2.13	0.144	
	Legume (L)	NA	NA	1.00	0.318	1.61	0.204	NA	NA	0.96	0.328	0.45	0.502	
	Selection (S)	0.00	0.959	0.00	0.966	0.00	0.966	0.00	0.951	0.00	0.978	0.00	0.978	
	SR x L	NA	NA	0.17	0.679	0.17	0.679	NA	NA	0.45	0.504	0.45	0.504	
	SR x S	0.66	0.415	0.82	0.365	0.66	0.418	0.68	0.411	0.91	0.339	0.74	0.389	
	L x S	NA	NA	0.24	0.625	0.40	0.525	NA	NA	0.22	0.635	0.40	0.528	
	SR x L x S	NA	NA	0.45	0.501	0.45	0.501	NA	NA	0.41	0.520	0.41	0.520	
	R^2 marginal/conditional	0.06/NA		0.09/NA		0.09/NA		0.06/NA		0.09/NA		0.09/NA		
**Volatile compound diversity**														
VOC Richness<br>Hill q0<br><br>Negative<br>binomial<br>N= 60	Veg. height	NA	NA	NA	NA	NA	NA	[3.47]	*0.062*	[3.47]	*0.062*	3.47	*0.062*	Decrease with increasing species richness. Plots with legumes has less compounds
	Species richness (SR)	0.37	0.544	0.37	0.544	1.58	0.209	**4.72**	**0.030**	**4.72**	**0.030**	**4.06**	**0.044**	
	Legume (L)	NA	NA	**6.51**	**0.011**	**5.30**	**0.021**	NA	NA	1.64	0.201	2.30	0.129	
	Selection (S)	[3.20]	*0.074*	[3.26]	*0.071*	[3.26]	*0.071*	[3.43]	*0.064*	[3.48]	*0.062*	[3.48]	*0.062*	
	SR x L	NA	NA	0.01	0.913	0.01	0.913	NA	NA	1.04	0.307	1.04	0.307	
	SR x S	0.00	0.962	0.00	0.999	0.06	0.804	0.01	0.934	0.00	0.995	0.07	0.795	
	L x S	NA	NA	2.12	0.145	2.06	0.151	NA	NA	2.16	0.142	2.09	0.148	
	SR x L x S	NA	NA	0.74	0.390	0.74	0.390	NA	NA	0.85	0.358	0.85	0.358	
	R^2 marginal/conditional	0.06/0.24		0.26/0.30		0.26/0.30								
VOC Shannon<br>Hill q1<br>sqrt<br>N= 60	Veg. height	NA	NA	NA	NA	NA	NA	0.42	0.515	0.42	0.515	0.42	0.515	Decrease with increasing species richness.
	Species richness (SR)	0.98	0.322	0.98	0.322	2.64	0.105	**3.97**	**0.048**	**3.97**	**0.048**	2.46	0.117	
	Legume (L)	NA	NA	**4.18**	**0.041**	2.53	0.112	NA	NA	1.90	0.168	2.41	0.121	
	Selection (S)	0.69	0.405	0.78	0.376	0.78	0.376	0.81	0.368	0.81	0.368	0.81	0.368	
	SR x L	NA	NA	0.16	0.692	0.16	0.692	NA	NA	0.46	0.499	0.46	0.499	
	SR x S	0.09	0.761	0.09	0.762	0.20	0.652	0.09	0.761	0.06	0.806	0.15	0.700	
	L x S	NA	NA	1.48	0.224	1.37	0.242	NA	NA	1.52	0.218	1.43	0.232	
	SR x L x S	NA	NA	1.18	0.277	1.18	0.277	NA	NA	1.34	0.247	1.34	0.247	
	R^2 marginal/conditional	0.06/NA		0.21/0.3		0.21/0.3		0.16/NA		0.24/0.31		0.24/0.31		
VOC Simpson<br>Hill q2	Veg. height	NA	NA	NA	NA	NA	NA	0.85	0.357	0.85	0.357	0.85	0.357	
	Species richness (SR)	1.82	0.177	1.82	0.177	2.99	*0.084*	[3.66]	*0.056*	[3.66]	*0.056*	2.57	0.109	

log10 N= 60	Legume (L)	NA	NA	**4.88**	**0.027**	[3.72]	*0.054*	NA	NA	2.20	0.138	[3.29]	*0.070*	
	Selection (S)	0.26	0.613	0.07	0.798	0.07	0.798	0.22	0.640	0.07	0.796	0.07	0.796	
	SR x L	NA	NA	0.74	0.389	0.74	0.389	NA	NA	0.81	0.367	0.81	0.367	
	SR x S	0.22	0.641	0.23	0.632	0.90	0.344	0.48	0.490	0.22	0.640	0.89	0.346	
	L x S	NA	NA	[3.09]	*0.079*	2.43	0.119	NA	NA	[3.11]	*0.078*	2.45	0.118	
	SR x L x S	NA	NA	0.39	0.530	0.39	0.530	NA	NA	0.42	0.516	0.42	0.516	
	R^2 marginal/conditional	0/NA		0.01/NA		0.01/NA		0/NA		0.01/NA		0.01/NA		

Table S5 Selection Experiment: Wald-chi-squared analysis of variance (ANOVA) results for the linear mixed models of *naïve* and *selected* *Plantago lanceolata* phytometers across a diversity gradient based on non-volatile untargeted metabolome diversity. The effects of vegetation height, plant species richness, selection history (*naïve* or *selected*) and legumes (presence or absence) on untargeted metabolome diversity were tested using mixed-effects models. Six models were run to disentangle the confounding effects: Model 1 examined species richness, selection treatment, and their interaction. Models 2 and 3 assessed legumes presence, either before or after species richness. Models 4-6 tested vegetation height, including it as a covariate. All models used plot nested within block as random effects. The table shows Chi-square (X^2) and p-values for fixed effects, with significant effects in bold (P< 0.05) and tendencies within brackets (P < 0.1). Data were transformed as needed to meet assumptions.

Variable	Factor	**Model 1** y~ SR* S+ (1| block/ plot)		**Model 2** y~ SR*L* S+ (1| block/ plot)		**Model 3** y~ L*SR*S+ (1| block/ plot)		**Model 4** y~ VG+SR*S+ (1| block/ plot)		**Model 5** y~ VG+SR*L*S+ (1| block/ plot)		**Model 6** y~ VG+L*SR* S+ (1| block/ plot)		Pattern
		X²	***P***	***X²***	***P***	***X²***	***P***	***X²***	***P***	***X²***	***P***	***X²***	***P***	
Metabolome richness Hill q0 negative binomial	Veg. height	NA	NA	NA	NA	NA	NA	1.95	0.162	1.95	0.162	1.95	0.162	
	Species richness (SR)	0.19	0.662	0.19	0.662	0.24	0.623	2.62	0.106	2.62	0.106	[2.96]	*0.085*	
	Legume (L)	NA	NA	0.08	0.773	0.03	0.857	NA	NA	0.73	0.394	0.38	0.538	
	Selection (S)	0.00	1.000	0.00	0.978	0.00	0.978	0.00	0.957	0.00	0.994	0.00	0.994	
	SR x L	NA	NA	[3.78]	*0.052*	[3.78]	*0.052*	NA	NA	2.06	0.151	2.06	0.151	
	SR x S	1.39	0.238	1.38	0.240	1.27	0.260	1.43	0.231	1.46	0.226	1.36	0.243	
	L x S	NA	NA	0.03	0.868	0.14	0.710	NA	NA	0.02	0.901	0.12	0.733	
	SR x L x S	NA	NA	1.11	0.293	1.11	0.293	NA	NA	1.18	0.278	1.18	0.278	
	$R^2_{marginal/conditional}$	0.06/0.24		0.26/0.30		0.26/0.30								
Shannon metabolome diversity Hill q1	Veg. height	NA	NA	NA	NA	NA	NA	0.01	0.937	0.01	0.937	0.01	0.937	Increase with increasing species richness only in *selected* phytometers
	Species richness (SR)	2.00	0.158	2.00	0.158	2.13	0.145	2.53	0.112	2.53	0.112	2.26	0.132	
	Legume (L)	NA	NA	0.35	0.555	0.22	0.642	NA	NA	0.06	0.809	0.32	0.569	
	Selection (S)	**4.04**	**0.044**	**4.25**	**0.039**	**4.25**	**0.039**	**4.14**	**0.042**	**4.23**	**0.040**	**4.23**	**0.040**	
	SR x L	NA	NA	1.90	0.169	1.90	0.169	NA	NA	1.68	0.195	1.68	0.195	
	SR x S	**6.35**	**0.012**	**6.71**	**0.010**	**5.80**	**0.016**	**6.39**	**0.011**	**6.70**	**0.010**	**5.78**	**0.016**	
	L x S	NA	NA	0.71	0.401	1.62	0.204	NA	NA	0.71	0.400	1.62	0.203	
	SR x L x S	NA	NA	0.74	0.388	0.74	0.388	NA	NA	0.74	0.389	0.74	0.389	
	$R^2_{marginal/conditional}$	0.11/0.13		0.15/0.16		0.15/0.16		0.12/NA		0.16/NA		0.16/NA		
Simpson metabolome diversity Hill q2	Veg. height	NA	NA	NA	NA	NA	NA	0.13	0.719	0.13	0.719	0.13	0.719	Increase with increasing species richness only in *selected* phytometers
	Species richness (SR)	0.34	0.559	0.34	0.559	0.39	0.530	0.79	0.373	0.79	0.373	0.62	0.431	
	Legume (L)	NA	NA	0.47	0.494	0.41	0.520	NA	NA	0.12	0.734	0.29	0.592	
	Selection (S)	1.57	0.210	1.72	0.189	1.72	0.189	1.64	0.200	1.72	0.190	1.72	0.190	
	SR x L	NA	NA	2.83	*0.092*	2.83	*0.092*	NA	NA	2.61	0.106	2.61	0.106	
	SR x S	**7.90**	**0.005**	**8.38**	**0.004**	**7.47**	**0.006**	**7.94**	**0.005**	**8.38**	**0.004**	**7.47**	**0.006**	
	L x S	NA	NA	0.48	0.489	1.39	0.239	NA	NA	0.48	0.490	1.39	0.239	
	SR x L x S	NA	NA	0.32	0.574	0.32	0.574	NA	NA	0.32	0.573	0.32	0.573	
	$R^2_{marginal/conditional}$	0.09/0.12		0.13/NA		0.13/NA		0.1/NA		0.13/NA		0.13/NA		

Table S6 Selection Experiment: Wald-chi-squared analysis of variance (ANOVA) results for the linear mixed models of *naïve* and *selected* *Plantago lanceolata* phytometers across a diversity gradient based on targeted defense metabolites. The effects of vegetation height, plant species richness, selection history (naïve or selected) and legumes (presence or absence) on untargeted metabolome diversity were tested using mixed-effects models. Six models were run to disentangle the confounding effects: Model 1 examined species richness, selection treatment, and their interaction. Models 2 and 3 assessed legumes presence, either before or after species richness. Models 4-6 tested vegetation height, including it as a covariate. All models used plot nested within block as random effects. The table shows Chi-square (X^2) and p-values for fixed effects, with significant effects in bold ($P< 0.05$) and tendencies within brackets ($P < 0.1$). Data were transformed as needed to meet assumptions.

Variable	Factor	Model 1 y~ SR* S+ (1| block/ plot)		Model 2 y~ SR*L* S+ (1| block/ plot)		Model 3 y~ L*SR*S+ (1| block/ plot)		Model 4 y~ VG+SR*S+ (1| block/ plot)		Model 5 y~ VG+SR*L*S+ (1| block/ plot)		Model 6 y~ VG+L*SR* S+ (1| block/ plot)		Pattern
		X^2	*P*	X^2	*P*	X^2	*P*	X^2	*P*	X^2	*P*	X^2	*P*	
**Defense hormones**														
Jasmonic acid (ng (gdw)-1) log10 N= 112	Veg. height	NA	NA	NA	NA	NA	NA	0.62	0.430	0.62	0.430	0.62	0.430	
	Species richness (SR)	0.00	0.979	0.00	0.979	0.06	0.811	0.56	0.454	0.56	0.454	0.53	0.466	
	Legume (L)	NA	NA	0.97	0.325	0.91	0.339	NA	NA	0.34	0.561	0.37	0.545	
	Selection (S)	1.79	0.181	1.65	0.199	1.65	0.199	1.62	0.203	1.57	0.210	1.57	0.210	
	SR x L	NA	NA	0.00	0.999	0.00	0.999	NA	NA	0.04	0.836	0.04	0.836	
	SR x S	0.02	0.881	0.03	0.857	0.05	0.819	0.03	0.865	0.03	0.852	0.05	0.819	
	L x S	NA	NA	0.07	0.786	0.05	0.817	NA	NA	0.06	0.812	0.04	0.843	
	SR x L x S	NA	NA	0.01	0.927	0.01	0.927	NA	NA	0.01	0.929	0.01	0.929	
	R^2 marginal/conditional	0.01/0.35		0.04/0.35		0.04/0.35		0.09/NA		0.1/NA		0.1/NA		
Jasmonic acid isoleucine (JA-Ile) N= 112	Veg. height	NA	NA	NA	NA	NA	NA	0.22	0.642	0.22	0.642	0.22	0.642	
	Species richness (SR)	1.30	0.254	1.30	0.254	3.17	*0.075*	2.86	*0.091*	2.86	*0.091*	2.25	0.134	
	Legume (L)	NA	NA	**4.58**	**0.032**	2.71	0.100	NA	NA	2.96	*0.086*	3.56	*0.059*	
	Selection (S)	[3.66]	*0.056*	[3.33]	*0.068*	[3.33]	*0.068*	[3.54]	*0.060*	[3.32]	*0.068*	3.32	*0.068*	
	SR x L	NA	NA	0.13	0.718	0.13	0.718	NA	NA	0.19	0.663	0.19	0.663	
	SR x S	1.38	0.239	1.55	0.213	1.33	0.249	1.38	0.239	1.54	0.215	1.31	0.253	
	L x S	NA	NA	0.14	0.713	0.35	0.552	NA	NA	0.15	0.702	0.38	0.540	
	SR x L x S	NA	NA	0.86	0.355	0.86	0.355	NA	NA	0.87	0.351	0.87	0.351	
	R^2 marginal/conditional	0.07/0.26		0.17/0.28		0.17/0.28		0.13/NA		0.2/NA		0.2/NA		
12-hydroxy-jasmonic acid (OH-JA) log10 N= 112	Veg. height	NA	NA	NA	NA	NA	NA	[3.10]	*0.078*	[3.10]	*0.078*	[3.10]	*0.078*	
	Species richness (SR)	1.35	0.246	1.35	0.246	1.01	0.316	0.11	0.738	0.11	0.738	0.11	0.746	
	Legume (L)	NA	NA	0.32	0.569	0.66	0.415	NA	NA	0.02	0.886	0.03	0.869	
	Selection (S)	0.15	0.695	0.17	0.676	0.17	0.676	0.17	0.685	0.16	0.689	0.16	0.689	
	SR x L	NA	NA	**6.76**	**0.009**	**6.76**	**0.009**	NA	NA	**6.44**	**0.011**	**6.44**	**0.011**	
	SR x S	0.31	0.575	0.35	0.556	0.75	0.387	0.34	0.559	0.41	0.521	0.83	0.361	
	L x S	NA	NA	2.06	0.151	1.66	0.198	NA	NA	2.02	0.155	1.60	0.206	
	SR x L x S	NA	NA	1.21	0.270	1.21	0.270	NA	NA	1.11	0.292	1.11	0.292	
	R^2 marginal/conditional	0.04/NA		0.17/NA		0.17/NA		0.07/0.19		0.18/NA		0.18/NA		

12-hydroxy-jasmonoyl-isoleucine (12OH-JA-Ile) glmer N= 112	Veg. height	NA	NA	NA	NA	NA	NA	0.09	0.770	0.09	0.770	0.09	0.770	
	Species richness (SR)	1.86	0.173	1.86	0.173	2.89	*0.089*	1.99	0.158	1.99	0.158	0.99	0.320	
	Legume (L)	NA	NA	1.68	0.195	0.64	0.422	NA	NA	1.51	0.219	2.52	0.112	
	Selection (S)	1.11	0.292	1.01	0.316	1.01	0.316	1.09	0.296	1.00	0.316	1.00	0.316	
	SR x L	NA	NA	0.49	0.482	0.49	0.482	NA	NA	0.45	0.501	0.45	0.501	
	SR x S	0.38	0.536	0.43	0.514	0.48	0.490	0.38	0.539	0.43	0.512	0.48	0.488	
	L x S	NA	NA	0.07	0.797	0.02	0.899	NA	NA	0.07	0.796	0.02	0.899	
	SR x L x S	NA	NA	[3.57]	*0.059*	[3.57]	*0.059*	NA	NA	[3.56]	*0.059*	[3.56]	*0.059*	
	$R^2_{marginal/conditional}$	0.11/NA		0.21/NA		0.21/NA		0.11/NA		0.21/NA		0.21/NA		
12-carboxy-jasmonoyl-L-isoleucine (COOH-JA-Ile) glmer N= 112	Veg. height	NA	NA	NA	NA	NA	NA	0.00	0.951	0.00	0.951	0.00	0.951	
	Species richness (SR)	0.68	0.410	0.68	0.410	0.70	0.401	0.86	0.353	0.86	0.353	0.85	0.355	
	Legume (L)	NA	NA	0.04	0.840	0.01	0.909	NA	NA	0.00	0.963	0.01	0.922	
	Selection (S)	0.17	0.682	0.16	0.692	0.16	0.692	0.14	0.709	0.14	0.708	0.14	0.708	
	SR x L	NA	NA	0.35	0.555	0.35	0.555	NA	NA	0.45	0.504	0.45	0.504	
	SR x S	0.84	0.358	0.70	0.403	**6.25**	**0.012**	0.89	0.345	0.75	0.388	**6.76**	**0.009**	
	L x S	NA	NA	**20.72**	**0.000**	**15.17**	**0.000**	NA	NA	**21.26**	**0.000**	**15.25**	**0.000**	
	SR x L x S	NA	NA	2.30	0.129	2.30	0.129	NA	NA	2.22	0.136	2.39	0.122	
	$R^2_{marginal/conditional}$	0.31/0.99		0.74/1		0.74/1		0.97/NA		0.77/1		0.79/1		
Total jasmonates N= 112	Veg. height	NA	NA	NA	NA	NA	NA	0.86	0.355	0.86	0.355	0.86	0.355	
	Species richness (SR)	1.04	0.307	1.04	0.307	1.10	0.293	0.27	0.604	0.27	0.604	0.00	1.000	
	Legume (L)	NA	NA	0.06	0.805	0.00	0.985	NA	NA	0.22	0.642	0.55	0.460	
	Selection (S)	1.59	0.207	1.57	0.211	1.57	0.211	1.61	0.205	1.84	0.175	1.84	0.175	
	SR x L	NA	NA	[2.86]	*0.091*	[2.86]	*0.091*	NA	NA	2.39	0.123	2.39	0.123	
	SR x S	0.31	0.578	0.34	0.558	0.59	0.441	0.31	0.575	0.35	0.554	0.60	0.437	
	L x S	NA	NA	1.00	0.318	0.75	0.388	NA	NA	1.00	0.317	0.75	0.388	
	SR x L x S	NA	NA	1.02	0.314	1.02	0.314	NA	NA	1.00	0.316	1.00	0.316	
	$R^2_{marginal/conditional}$	0.05/NA		0.13/NA		0.13/NA		0.04/0.24		0.14/NA		0.14/NA		
Abscisic acid log10 N =112	Veg. height	NA	NA	NA	NA	NA	NA	**9.23**	**0.002**	**9.23**	**0.002**	**9.23**	**0.002**	Decrease with increasing vegetation height
	Species richness (SR)	**3.44**	***0.048***	**3.44**	***0.048***	[3.11]	*0.078*	0.12	0.733	0.12	0.733	0.05	0.825	
	Legume (L)	NA	NA	0.03	0.873	0.35	0.555	NA	NA	2.51	0.113	2.58	0.108	
	Selection (S)	0.03	0.864	0.03	0.858	0.03	0.858	0.11	0.736	0.07	0.790	0.07	0.790	
	SR x L	NA	NA	[2.87]	*0.090*	2.87	*0.090*	NA	NA	1.34	0.248	1.34	0.248	
	SR x S	1.68	0.195	1.70	0.192	1.22	0.269	1.56	0.211	1.88	0.171	1.38	0.240	
	L x S	NA	NA	0.85	0.357	1.33	0.249	NA	NA	0.77	0.380	1.26	0.261	
	SR x L x S	NA	NA	1.30	0.254	1.30	0.254	NA	NA	1.30	0.254	1.30	0.254	
	$R^2_{marginal/conditional}$	0.11/NA		0.15/0.25		0.15/0.25		0.29/NA		0.3/NA		0.3/NA		
Salicylic acid Log1p N=112	Veg. height	NA	NA	NA	NA	NA	NA	[3.28]	0.070	[3.28]	0.070	[3.28]	0.070	Decreased with increasing diversity.
	Species richness (SR)	**7.06**	**0.005**	**7.06**	**0.005**	**7.91**	**0.004**	**6.05**	**0.014**	**6.05**	**0.014**	**6.27**	**0.012**	
	Legume (L)	NA	NA	0.33	0.566	0.019	0.891	NA	NA	2.58	0.108	2.58	*0.108*	
	Selection (S)	0.02	0.877	0.04	0.849	0.036	0.849	0.001	0.965	0.11	0.918	0.11	0.918	
	SR x L	NA	NA	[3.02]	0.082	[3.01]	0.082	NA	NA	0.97	0.323	0.97	0.323	
	SR x S	2.37	0.123	2.27	0.131	0.01	0.941	2.41	0.120	2.10	0.147	2.14	0.143	
	L x S	NA	NA	0.04	0.833	2.315	0.128	NA	NA	0.04	0.839	0.01	0.946	
	SR x L x S	NA	NA	1.75	0.185	1.75	0.185	NA	NA	1.56	0.210	1.56	0.210	
	$R^2_{marginal/conditional}$	0.27/0.46		0.31/0.45		0.31/0.45		0.31/0.44		0.35/0.46		0.35/0.46		
**Iridoid glycosides**														
	Veg. height	NA	NA	NA	NA	NA	NA	**8.02**	**0.005**	**8.02**	**0.005**	**8.02**	**0.005**	

Aucubin N=107	Species richness (SR)	0.16	0.693	0.16	0.693	0.37	0.543	**9.45**	**0.002**	**9.45**	**0.002**	**10.36**	**0.001**	Considering the veg. height, concertation decreased with increasing SR. Veg. height increased concentration
	Legume (L)	NA	NA	1.29	0.257	1.07	0.301	NA	NA	1.05	0.305	0.14	0.707	
	Selection (S)	0.79	0.374	0.94	0.333	0.94	0.333	1.18	0.277	1.12	0.291	1.12	0.291	
	SR x L	NA	NA	2.52	0.112	2.52	0.112	NA	NA	0.73	0.394	0.73	0.394	
	SR x S	0.99	0.319	0.91	0.341	0.83	0.361	1.09	0.297	0.97	0.325	0.88	0.348	
	L x S	NA	NA	0.02	0.885	0.09	0.761	NA	NA	0.03	0.861	0.12	0.730	
	SR x L x S	NA	NA	[3.34]	*0.068*	[3.34]	*0.068*	NA	NA	[3.48]	*0.062*	[3.48]	*0.062*	
	$R^2_{marginal/conditional}$	0.02/0.13		0.09/0.15		0.09/0.15		0.19/NA		0.23/NA		0.23/NA		
Catalpol N=107	Veg. height	NA	NA	NA	NA	NA	NA	2.60	0.107	2.60	0.107	2.60	0.107	
	Species richness (SR)	0.31	0.579	0.31	0.579	0.34	0.560	0.12	0.726	0.12	0.726	0.41	0.523	
	Legume (L)	NA	NA	0.03	0.855	0.00	0.964	NA	NA	2.09	0.149	1.80	0.179	
	Selection (S)	0.00	0.993	0.00	0.996	0.00	0.996	0.01	0.935	0.00	1.000	0.00	1.000	
	SR x L	NA	NA	[2.91]	*0.088*	[2.91]	*0.088*	NA	NA	[2.94]	*0.087*	[2.94]	*0.087*	
	SR x S	0.97	0.326	0.84	0.361	0.60	0.440	0.96	0.327	0.73	0.392	0.54	0.462	
	L x S	NA	NA	0.60	0.440	0.84	0.361	NA	NA	0.39	0.530	0.59	0.443	
	SR x L x S	NA	NA	0.44	0.506	0.44	0.506	NA	NA	0.28	0.595	0.28	0.595	
	$R^2_{marginal/conditional}$	0.02/NA		0.07/NA		0.07/NA		0.05/NA		0.11/NA		0.11/NA		
**Phenylpropanoid glycosides**														
Verbascoside N=107	Veg. height	NA	NA	NA	NA	NA	NA	0.15	0.701	0.15	0.701	0.15	0.701	Decreased as SR increased. Naïve phytometers in communities without legumes did not vary across the diversity gradient
	Species richness (SR)	2.38	0.123	2.38	0.123	2.21	0.137	**4.65**	**0.031**	**4.65**	**0.031**	**3.88**	**0.049**	
	Legume (L)	NA	NA	0.75	0.386	0.92	0.338	NA	NA	0.01	0.925	0.77	0.379	
	Selection (S)	1.19	0.276	1.29	0.256	1.29	0.256	1.34	0.246	1.35	0.244	1.35	0.244	
	SR x L	NA	NA	**4.92**	**0.027**	**4.92**	**0.027**	NA	NA	**4.39**	**0.036**	**4.39**	**0.036**	
	SR x S	0.14	0.709	0.14	0.706	0.15	0.698	0.15	0.699	0.14	0.712	0.14	0.704	
	L x S	NA	NA	0.01	0.925	0.00	0.978	NA	NA	0.01	0.926	0.00	0.978	
	SR x L x S	NA	NA	**4.49**	**0.034**	**4.49**	**0.034**	NA	NA	**4.41**	**0.036**	**4.41**	**0.036**	
	$R^2_{marginal/conditional}$	0.1/NA		0.25/0.34		0.25/0.34		0.17/NA		0.28/NA		0.28/NA		
Plantamajoside N=112	Veg. height	NA	NA	NA	NA	NA	NA	2.35	0.125	2.35	0.125	2.35	0.125	Decreased as SR increased.
	Species richness (SR)	**7.14**	**0.008**	**7.14**	**0.008**	**7.08**	**0.008**	**4.96**	**0.026**	**4.96**	**0.026**	**5.67**	**0.017**	
	Legume (L)	NA	NA	0.27	0.604	0.33	0.568	NA	NA	0.73	0.394	0.02	0.892	
	Selection (S)	0.24	0.625	0.28	0.599	0.28	0.599	0.22	0.640	0.26	0.609	0.26	0.609	
	SR x L	NA	NA	1.36	0.244	1.36	0.244	NA	NA	0.94	0.333	0.94	0.333	
	SR x S	0.09	0.761	0.13	0.723	0.32	0.573	0.09	0.764	0.13	0.720	0.33	0.568	
	L x S	NA	NA	1.18	0.277	0.99	0.320	NA	NA	1.21	0.271	1.01	0.314	
	SR x L x S	NA	NA	3.11	*0.078*	3.11	*0.078*	NA	NA	[3.08]	*0.079*	[3.08]	*0.079*	
	$R^2_{marginal/conditional}$	0.12/NA		0.2/NA		0.2/NA		0.13/NA		0.2/NA		0.2/NA		

Table S7 **Community History Experiment: Wald-chi-squared analysis of variance (ANOVA) results for the linear mixed models of selected *Plantago lanceolata* phytometers across a diversity gradient in different community history environments based on volatile organic compounds profiles.** The effects of vegetation height, species richness, experimental environment (*S+P+*, *S+P-*, *S-P-*) and legumes (presence or absence) on volatile organic compounds diversity were tested using mixed-effects models. *Community History Experiment* compared the metabolomic profiles of *selected* phytometers grown in different environment treatments based on the ΔBEF experiment established in 2016 (Vogel et al., 2019). Six models were run to disentangle the confounding effects: Model 1 examined species richness, environment treatment, and their interaction. Models 2 and 3 assessed legumes presence, either before or after species richness. Models 4-6 tested vegetation height, including it as a covariate. All models used plot nested within block as random effects. The table shows Chi-square (X^2) and p-values for fixed effects, with significant effects in bold ($P< 0.05$) and tendencies within brackets ($P < 0.1$). Data were transformed as needed to meet assumptions. N= 86

Variable	Factor	Model 1 y~ SR* E+ (1| block/ plot)		Model 2 y~ SR*L* E+ (1| block/ plot)		Model 3 y~ L*SR*E+ (1| block/ plot)		Model 4 y~ VG+SR*E+ (1| block/ plot)		Model 5 y~ VG+SR*L*E+ (1| block/ plot)		Model 6 y~ VG+L*SR* E+ (1| block/ plot)		Pattern
		X²	***P***	***X²***	***P***	***X²***	***P***	***X²***	***P***	***X²***	***P***	***X²***	***P***	
**Emission of volatile organic compounds**														
Aromatics	Veg. height	NA	NA	NA	NA	NA	NA	0.03	0.852	0.03	0.852	0.03	0.852	Decreased with increasing species richness
	Species richness (SR)	3.07	*0.080*	3.07	*0.080*	3.26	*0.071*	**4.68**	**0.031**	**4.68**	**0.031**	**4.52**	**0.034**	
	Legume (L)	NA	NA	0.24	0.628	0.05	0.817	NA	NA	0.02	0.885	0.18	0.670	
	Environment (E)	2.38	0.304	2.40	0.301	2.40	0.301	3.53	0.171	3.69	0.158	3.69	0.158	
	SR x L	NA	NA	1.36	0.244	1.36	0.244	NA	NA	1.39	0.239	1.39	0.239	
	SR x E	4.27	0.118	4.64	*0.098*	2.61	0.271	3.56	0.168	3.94	0.140	1.92	0.383	
	L x E	NA	NA	3.94	0.140	5.97	*0.051*	NA	NA	4.12	0.128	**6.14**	**0.046**	
	SR x L x E	NA	NA	2.78	0.249	2.78	0.249	NA	NA	3.08	0.214	3.08	0.214	
	R^2 marginal/conditional	0.16/0.29		0.25/NA		0.25/NA		0.19/0.37		0.24/0.38		0.24/0.38		
Green leaf volatiles	Veg. height	NA	NA	NA	NA	NA	NA	1.04	0.308	1.04	0.308	1.04	0.308	Higher in plots with legumes
	Species richness (SR)	0.92	0.337	0.92	0.337	0.26	0.610	0.11	0.744	0.11	0.744	0.47	0.493	
	Legume (L)	NA	NA	**4.12**	**0.042**	**4.78**	**0.029**	NA	NA	**4.11**	**0.043**	3.75	*0.053*	
	Environment (E)	2.37	0.306	2.99	0.224	2.99	0.224	3.00	0.223	2.79	0.248	2.79	0.248	
	SR x L	NA	NA	3.51	*0.061*	3.51	*0.061*	NA	NA	3.72	*0.054*	3.72	*0.054*	
	SR x E	0.49	0.782	1.09	0.579	0.90	0.638	0.62	0.734	1.02	0.601	0.82	0.663	
	L x E	NA	NA	0.03	0.984	0.23	0.892	NA	NA	0.04	0.979	0.24	0.887	
	SR x L x E	NA	NA	1.08	0.584	1.08	0.584	NA	NA	0.94	0.625	0.94	0.625	
	R^2 marginal/conditional	0.05/NA		0.16/NA		0.16/NA		0.06/NA		0.16/NA		0.16/NA		
Monoterpenes	Veg. height	NA	NA	NA	NA	NA	NA	0.58	0.448	0.58	0.448	0.58	0.448	Decreased with increasing species richness only in
	Species richness (SR)	2.08	0.149	2.08	0.149	2.08	0.149	**5.11**	**0.024**	**5.11**	**0.024**	**6.13**	**0.013**	
	Legume (L)	NA	NA	0.00	0.994	0.00	0.954	NA	NA	1.10	0.293	0.09	0.764	

	Environment (E)	0.26	0.879	0.26	0.879	0.26	0.879	0.02	0.991	0.00	0.998	0.00	0.998	environments with soil history
	SR x L	NA	NA	0.83	0.361	0.83	0.361	NA	NA	1.61	0.205	1.61	0.205	
	SR x E	**8.53**	**0.014**	**9.25**	**0.010**	**7.23**	**0.027**	**7.31**	**0.026**	**8.38**	**0.015**	**6.26**	**0.044**	
	L x E	NA	NA	2.69	0.260	4.71	*0.095*	NA	NA	2.25	0.325	4.37	0.113	
	SR x L x E	NA	NA	2.83	0.243	2.83	0.243	NA	NA	1.90	0.388	1.90	0.388	
	R^2 marginal/conditional	0.14/NA		0.19/NA		0.19/NA		0.14/0.22		0.22/NA		0.22/NA		
Sesquiterpenes	Veg. height	NA	NA	NA	NA	NA	NA	1.75	0.186	1.75	0.186	1.75	0.186	Decreased with species richness when considering the surrounding vegetation height
	Species richness (SR)	2.03	0.154	2.03	0.154	2.07	0.151	**6.84**	**0.009**	**6.84**	**0.009**	**7.27**	**0.007**	
	Legume (L)	NA	NA	0.25	0.615	0.21	0.644	NA	NA	0.47	0.493	0.04	0.837	
	Environment (E)	0.53	0.767	0.58	0.749	0.58	0.749	0.80	0.670	0.78	0.675	0.78	0.675	
	SR x L	NA	NA	0.62	0.430	0.62	0.430	NA	NA	1.95	0.163	1.95	0.163	
	SR x E	4.08	0.130	3.96	0.138	2.69	0.261	3.74	0.154	4.00	0.136	2.58	0.275	
	L x E	NA	NA	6.69	**0.035**	7.96	**0.019**	NA	NA	5.25	*0.072*	**6.67**	**0.036**	
	SR x L x E	NA	NA	4.79	*0.091*	4.79	*0.091*	NA	NA	2.61	0.272	2.61	0.272	
	R^2 marginal/conditional	0.09/0.17		0.2/0.25		0.2/0.25		0.17/NA		0.25/NA		0.25/NA		
Others	Veg. height	NA	NA	NA	NA	NA	NA	0.33	0.567	0.33	0.567	0.33	0.567	Decreased with species richness
	Species richness (SR)	**4.68**	**0.030**	**4.68**	**0.030**	**5.01**	**0.025**	**8.44**	**0.004**	**8.44**	**0.004**	**8.82**	**0.003**	
	Legume (L)	NA	NA	0.33	0.563	0.01	0.941	NA	NA	0.46	0.499	0.07	0.788	
	Environment (E)	1.17	0.556	1.14	0.564	1.14	0.564	2.01	0.366	2.28	0.319	2.28	0.319	
	SR x L	NA	NA	0.18	0.672	0.18	0.672	NA	NA	0.00	0.987	0.00	0.987	
	SR x E	2.71	0.258	2.48	0.289	1.74	0.419	1.79	0.409	1.82	0.402	1.05	0.591	
	L x E	NA	NA	2.26	0.324	3.00	0.223	NA	NA	1.89	0.388	2.67	0.264	
	SR x L x E	NA	NA	1.12	0.571	1.12	0.571	NA	NA	1.33	0.514	1.33	0.514	
	R^2 marginal/conditional	0.15/NA		0.19/NA		0.19/NA		0.2/NA		0.23/NA		0.23/NA		
Total emission	Veg. height	NA	NA	NA	NA	NA	NA	0.89	0.345	0.89	0.345	0.89	0.345	Tendency to decrease with increasing species richness
	Species richness (SR)	3.70	*0.054*	3.70	*0.054*	2.51	0.113	2.94	*0.087*	2.94	*0.087*	**4.39**	**0.036**	
	Legume (L)	NA	NA	1.58	0.209	2.77	*0.096*	NA	NA	3.33	*0.068*	1.88	0.170	
	Environment (E)	1.09	0.579	1.39	0.499	1.39	0.499	0.98	0.612	0.87	0.649	0.87	0.649	
	SR x L	NA	NA	0.51	0.475	0.51	0.475	NA	NA	0.93	0.335	0.93	0.335	
	SR x E	1.92	0.384	2.64	0.267	1.93	0.381	1.91	0.385	2.34	0.311	1.62	0.444	
	L x E	NA	NA	0.34	0.842	1.05	0.591	NA	NA	0.45	0.798	1.17	0.558	
	SR x L x E	NA	NA	1.56	0.459	1.56	0.459	NA	NA	1.00	0.607	1.00	0.607	
	R^2 marginal/conditional	0.08/NA		0.13/NA		0.13/NA		0.08/NA		0.14/NA		0.14/NA		
**Volatile compound diversity**														
VOC richness Hill q0	Veg. height	NA	NA	NA	NA	NA	NA	0.08	0.778	0.08	0.778	0.08	0.778	
	Species richness (SR)	1.57	0.210	1.57	0.210	2.17	0.141	3.50	*0.061*	3.50	*0.061*	3.33	*0.068*	
	Legume (L)	NA	NA	0.90	0.342	0.31	0.581	NA	NA	0.06	0.811	0.23	0.634	
	Environment (E)	0.50	0.780	0.54	0.763	0.54	0.763	0.34	0.842	0.37	0.829	0.37	0.829	
	SR x L	NA	NA	0.35	0.553	0.35	0.553	NA	NA	0.83	0.363	0.83	0.363	

	SR x E	1.98	0.371	1.84	0.398	2.25	0.324	1.84	0.398	1.75	0.417	2.05	0.359	
	L x E	NA	NA	1.68	0.432	1.27	0.531	NA	NA	1.21	0.547	0.90	0.637	
	SR x L x E	NA	NA	0.53	0.766	0.53	0.766	NA	NA	0.56	0.754	0.56	0.754	
	$R^2_{marginal/conditional}$	0.08/NA		0.1/NA		0.1/NA		0.09/NA		0.1/NA		0.1/NA		
VOC Shannon Diversity Hill q1	Veg. height	NA	NA	NA	NA	NA	NA	0.01	0.925	0.00	0.967	0.00	0.967	Decreased with increasing species richness only in phytometers that grew in environments with soil history (S+P+, S+P-), but remained similar across the species richness gradient in environments without history
	Species richness (SR)	0.26	0.612	0.18	0.669	0.41	0.520	0.46	0.495	0.26	0.612	0.23	0.633	
	Legume (L)	NA	NA	0.79	0.374	0.56	0.455	NA	NA	0.72	0.395	0.75	0.385	
	Environment (E)	2.50	0.287	1.63	0.442	1.63	0.442	3.24	0.198	1.69	0.429	1.69	0.429	
	SR x L	NA	NA	0.06	0.814	0.06	0.814	NA	NA	0.08	0.773	0.08	0.773	
	SR x E	3.13	0.209	2.47	0.290	2.21	0.332	**5.55**	**0.048**	**5.37**	**0.049**	**2.08**	**0.354**	
	L x E	NA	NA	0.03	0.983	0.30	0.861	NA	NA	0.03	0.983	0.33	0.849	
	SR x L x E	NA	NA	0.99	0.610	0.99	0.610	NA	NA	1.00	0.605	1.00	0.605	
	$R^2_{marginal/conditional}$	0.08/NA		0.1/NA		0.1/NA		0.09/NA		0.1/NA		0.1/NA		
VOC Simpson Diversity Hill q2	Veg. height	NA	NA	NA	NA	NA	NA	0.01	0.932	0.01	0.932	0.01	0.932	
	Species richness (SR)	0.07	0.786	0.07	0.786	0.27	0.601	0.07	0.786	0.07	0.786	0.08	0.773	
	Legume (L)	NA	NA	1.04	0.307	0.84	0.359	NA	NA	1.13	0.288	1.12	0.290	
	Environment (E)	3.28	0.194	3.38	0.184	3.38	0.184	3.70	0.157	3.36	0.186	3.36	0.186	
	SR x L	NA	NA	0.08	0.784	0.08	0.784	NA	NA	0.10	0.746	0.10	0.746	
	SR x E	2.17	0.338	2.02	0.364	1.40	0.498	1.82	0.402	1.92	0.383	1.30	0.521	
	L x E	NA	NA	0.40	0.818	1.03	0.599	NA	NA	0.40	0.818	1.02	0.601	
	SR x L x E	NA	NA	1.50	0.472	1.50	0.472	NA	NA	1.56	0.458	1.56	0.458	
	$R^2_{marginal/conditional}$	0.05/0.36		0.14/NA		0.14/NA		0.05/0.34		0.1/0.36		0.1/0.36		

Table S8. **Community History Experiment: Wald-chi-squared analysis of variance (ANOVA) results for the linear mixed models of *selected Plantago lanceolata* phytometers across a diversity gradient in different community history environments based on untargeted metabolome diversity.** The effects of vegetation height, species richness, environment treatment (*S+P+*, *S+P-*, *S-P-*) and legumes (presence or absence) on metabolome diversity were tested using mixed-effects models. *Community History Experiment* compared the metabolomic profiles of *selected* phytometers grown in different environment treatments based on the ΔBEF experiment established in 2016 (Vogel et al., 2019). Six models were run to disentangle the confounding effects: Model 1 examined species richness, environment treatment, and their interaction. Models 2 and 3 assessed legumes presence, either before or after species richness. Models 4-6 tested vegetation height, including it as a covariate. All models used plot nested within block as random effects. The table shows Chi-square (X^2) and P-values for fixed effects, with significant effects in bold (P< 0.05) and tendencies within brackets (P < 0.1). Data were transformed as needed to meet assumptions.

Variable	Factor	**Model 1** y~ SR* E+ (1| block/ plot)		**Model 2** y~ SR*L* E+ (1| block/ plot)		**Model 3** y~ L*SR*E+ (1| block/ plot)		**Model 4** y~ VG+SR*E+ (1| block/ plot)		**Model 5** y~ VG+SR*L*E+ (1| block/ plot)		**Model 6** y~ VG+L*SR* E+ (1| block/ plot)		Pattern
		X^2	P	X^2	P	X^2	P	X^2	P	X^2	P	X^2	P	
Metabolome richness Hill q0 negative binomial	Veg. height	NA	NA	NA	NA	NA	NA	0.61	0.437	0.61	0.437	0.61	0.437	
	Species richness (SR)	0.02	0.888	0.02	0.888	0.02	0.884	0.14	0.713	0.14	0.713	0.15	0.703	
	Legume (L)	NA	NA	0.00	0.971	0.00	0.998	NA	NA	0.22	0.638	0.21	0.646	
	Environment (E)	0.42	0.809	0.42	0.810	0.42	0.810	0.69	0.707	0.72	0.696	0.72	0.696	
	SR x L	NA	NA	3.12	0.078	3.12	0.078	NA	NA	3.20	0.074	3.20	0.074	
	SR x E	0.38	0.827	0.34	0.842	0.40	0.819	0.50	0.777	0.45	0.800	0.48	0.787	
	L x E	NA	NA	0.56	0.756	0.50	0.778	NA	NA	0.44	0.803	0.41	0.817	
	SR x L x E	NA	NA	1.63	0.442	1.63	0.442	NA	NA	1.38	0.501	1.38	0.501	
	R^2 marginal/conditional													
Shannon metabolome diversity Hill q1	Veg. height	NA	NA	NA	NA	NA	NA	0.05	0.831	0.05	0.831	0.05	0.831	Increased with increasing species richness only in selected phytometers that grew in the environment of origin
	Species richness (SR)	0.03	0.859	0.03	0.859	0.04	0.840	0.34	0.560	0.34	0.560	0.31	0.575	
	Legume (L)	NA	NA	0.06	0.801	0.05	0.816	NA	NA	0.00	0.998	0.03	0.872	
	Environment (E)	1.69	0.430	1.64	0.440	1.64	0.440	1.43	0.488	1.43	0.488	1.43	0.488	
	SR x L	NA	NA	1.60	0.207	1.60	0.207	NA	NA	1.54	0.214	1.54	0.214	
	SR x E	**10.36**	**0.006**	**10.43**	**0.005**	**9.55**	**0.008**	**10.60**	**0.005**	**10.43**	**0.005**	**9.45**	**0.009**	
	L x E	NA	NA	0.13	0.935	1.01	0.603	NA	NA	0.14	0.935	1.11	0.574	
	SR x L x E	NA	NA	0.88	0.645	0.88	0.645	NA	NA	0.87	0.647	0.87	0.647	
	R^2 marginal/conditional	0.08/0.16		0.1/0.16		0.1/0.16		0.08/NA		0.1/0.16		0.1/0.16		
Simpson metabolome diversity Hill q2	Veg. height	NA	NA	NA	NA	NA	NA	0.00	0.971	0.00	0.971	0.00	0.971	Increased with increasing species richness only in selected phytometers that grew in the environment of origin
	Species richness (SR)	0.22	0.638	0.22	0.638	0.21	0.646	0.63	0.428	0.63	0.428	0.65	0.419	
	Legume (L)	NA	NA	0.00	0.989	0.01	0.921	NA	NA	0.04	0.839	0.02	0.900	
	Environment (E)	1.62	0.446	1.62	0.446	1.62	0.446	1.64	0.440	1.68	0.433	1.68	0.433	
	SR x L	NA	NA	2.44	0.118	2.44	0.118	NA	NA	2.30	0.130	2.30	0.130	
	SR x E	**8.23**	**0.016**	**8.39**	**0.015**	**7.78**	**0.020**	**7.96**	**0.019**	**8.16**	**0.017**	**7.49**	**0.024**	
	L x E	NA	NA	0.14	0.933	0.74	0.689	NA	NA	0.12	0.942	0.79	0.673	
	SR x L x E	NA	NA	1.34	0.511	1.34	0.511	NA	NA	1.27	0.531	1.27	0.531	
	R^2 marginal/conditional	0.07/0.14		0.1/0.14		0.1/0.14		0.07/NA		0.1/0.15		0.1/0.15		

Table S9. **Community History Experiment: Wald-chi-squared analysis of variance (ANOVA) results for the linear mixed models of *selected Plantago lanceolata* phytometers across a diversity gradient in different community history environments based on targeted defense compounds**. The effects of vegetation height, species richness, *experimental* environment *(S+P+, S+P-, S-P-)* and legumes (presence or absence) on targeted defense compounds were tested using mixed-effects models. *Community History Experiment* compared the metabolomic profiles of *selected* phytometers grown in different environment treatments based on the ΔBEF experiment established in 2016 (Vogel et al., 2019). Six models were run to disentangle the confounding effects: Model 1 examined species richness, environment treatment, and their interaction. Models 2 and 3 assessed legumes presence, either before or after species richness. Models 4-6 tested vegetation height, including it as a covariate. All models used plot nested within block as random effects. The table shows Chi-square (X^2) and P-values for fixed effects, with significant effects in bold (P< 0.05) and tendencies within brackets (P < 0.1). Data were transformed as needed to meet assumptions. N = 163.

Variable	Factor	Model 1		Model 2		Model 3		Model 4		Model 5		Model 6		Pattern
		y~ SR* E+ (1| block/ plot)		y~ SR*L* E+ (1| block/ plot)		y~ L*SR*E+ (1| block/ plot)		y~ VG+SR*E+ (1| block/ plot)		y~ VG+SR*L*E+ (1| block/ plot)		y~ VG+L*SR* E+ (1| block/ plot)		
		X^2	***P***	***X^2***	***P***	***X^2***	***P***	***X^2***	***P***	***X^2***	***P***	***X^2***	***P***	
**Defense hormones**														
Jasmonic acid (ng (gdw)-1) log10	Veg. height	NA	NA	NA	NA	NA	NA	[3.38]	*0.066*	[3.38]	*0.066*	[3.38]	*0.066*	
	Species richness (SR)	0.03	0.871	0.03	0.871	0.04	0.833	1.04	0.309	1.04	0.309	0.99	0.320	
	Legume (L)	NA	NA	0.05	0.820	0.03	0.854	NA	NA	0.00	0.955	0.05	0.827	
	Environment (E)	2.09	0.352	2.11	0.348	2.11	0.348	2.07	0.356	2.07	0.356	2.07	0.356	
	SR x L	NA	NA	0.45	0.503	0.45	0.503	NA	NA	0.44	0.508	0.44	0.508	
	SR x E	[5.19]	*0.075*	[5.19]	*0.075*	[5.19]	*0.075*	4.30	0.116	4.30	0.117	4.46	0.107	
	L x E	NA	NA	1.47	0.480	1.19	0.553	NA	NA	0.74	0.690	0.58	0.750	
	SR x L x E	NA	NA	2.12	0.346	2.12	0.346	NA	NA	1.52	0.467	1.52	0.467	
	R^2 marginal/conditional	0.03/0.32		0.06/0.33		0.06/0.33		0.12/NA		0.14/NA		0.14/NA		
Jasmonic acid isoleucine (JA-Ile)	Veg. height	NA	NA	NA	NA	NA	NA	0.95	0.330	0.95	0.330	0.95	0.330	
	Species richness (SR)	1.75	0.185	1.75	0.185	1.88	0.171	[3.36]	*0.067*	[3.36]	*0.067*	3.36	*0.067*	
	Legume (L)	NA	NA	0.15	0.699	0.03	0.872	NA	NA	0.02	0.889	0.01	0.910	
	Environment (E)	0.67	0.717	0.69	0.708	0.69	0.708	0.49	0.782	0.50	0.779	0.50	0.779	
	SR x L	NA	NA	1.08	0.299	1.08	0.299	NA	NA	0.88	0.348	0.88	0.348	
	SR x E	**6.29**	**0.043**	**6.23**	**0.044**	**10.03**	**0.007**	[5.53]	*0.063*	[5.56]	*0.062*	**9.11**	**0.011**	
	L x E	NA	NA	**6.49**	**0.039**	2.70	0.260	NA	NA	[5.88]	*0.053*	2.33	0.312	
	SR x L x E	NA	NA	3.68	0.159	3.68	0.159	NA	NA	3.18	0.204	3.18	0.204	
	R^2 marginal/conditional	0.07/0.22		0.12/0.25		0.12/0.25		0.11/NA		0.14/0.26		0.14/0.26		
	Veg. height	NA	NA	NA	NA	NA	NA	0.14	0.708	0.14	0.708	0.14	0.708	Decreased with increasing species
	Species richness (SR)	2.05	0.152	2.05	0.152	[3.27]	*0.071*	2.52	0.113	2.52	0.113	[2.97]	*0.085*	

12-hydroxy-jasmonic acid (OH-JA)	Legume (L)	NA	NA	1.89	0.169	0.67	0.413	NA	NA	1.30	0.254	0.85	0.356	richness in communities without legumes
	Environment (E)	1.58	0.455	1.65	0.437	1.65	0.437	1.27	0.531	1.69	0.430	1.69	0.430	
	SR x L	NA	NA	**7.96**	**0.005**	**7.96**	**0.005**	NA	NA	**7.98**	**0.005**	**7.98**	**0.005**	
	SR x E	3.05	0.217	3.25	0.197	3.13	0.209	2.86	0.239	3.20	0.202	3.10	0.212	
	L x E	NA	NA	0.96	0.618	1.08	0.583	NA	NA	0.94	0.624	1.05	0.593	
	SR x L x E	NA	NA	0.87	0.647	0.87	0.647	NA	NA	0.87	0.648	0.87	0.648	
	R^2 marginal/conditional	0.07/0.2		0.15/NA		0.15/NA		0.06/0.19		0.15/NA		0.15/NA		
12-hydroxy-jasmonoyl-isoleucine (12OH-JA-Ile)	Veg. height	NA	NA	NA	NA	NA	NA	1.17	0.279	1.17	0.279	1.17	0.279	Decreased with increasing species richness only in S+P-
	Species richness (SR)	[3.65]	*0.056*	[3.65]	*0.056*	**3.87**	**0.049**	**5.94**	**0.015**	**5.94**	**0.015**	**5.86**	**0.015**	
	Legume (L)	NA	NA	0.22	0.636	0.00	0.964	NA	NA	0.01	0.926	0.09	0.767	
	Environment (E)	0.46	0.793	0.46	0.795	0.46	0.795	0.15	0.926	0.15	0.927	0.15	0.927	
	SR x L	NA	NA	0.22	0.637	0.22	0.637	NA	NA	0.21	0.650	0.21	0.650	
	SR x E	**7.25**	**0.027**	**7.16**	**0.028**	**7.92**	**0.019**	**6.53**	**0.038**	**6.52**	**0.038**	**7.21**	**0.027**	
	L x E	NA	NA	2.78	0.249	2.03	0.363	NA	NA	2.16	0.340	1.46	0.481	
	SR x L x E	NA	NA	1.42	0.492	1.42	0.492	NA	NA	1.17	0.558	1.17	0.558	
	R^2 marginal/conditional	0.12/0.29		0.14/0.3		0.14/0.3		0.18/NA		0.19/NA		0.19/NA		
12-carboxy-jasmonoyl-L-isoleucine (COOH-JA-Ile) glmer	Veg. height	NA	NA	NA	NA	NA	NA	**5.67**	**0.017**	**5.67**	**0.017**	**5.67**	**0.017**	Decreased with increasing vegetation height
	Species richness (SR)	0.78	0.378	0.78	0.378	0.77	0.380	2.47	0.116	2.47	0.116	2.07	0.150	
	Legume (L)	NA	NA	0.01	0.908	0.02	0.893	NA	NA	0.63	0.426	1.03	0.309	
	Environment (E)	2.51	0.284	2.51	0.285	2.51	0.285	0.30	0.861	0.20	0.904	0.20	0.904	
	SR x L	NA	NA	0.01	0.940	0.01	0.940	NA	NA	0.00	0.966	0.00	0.966	
	SR x E	0.53	0.767	0.54	0.763	0.50	0.778	0.26	0.878	0.27	0.875	0.36	0.834	
	L x E	NA	NA	**11.42**	**0.003**	**11.46**	**0.003**	NA	NA	**7.63**	**0.022**	**7.54**	**0.023**	
	SR x L x E	NA	NA	1.64	0.44	1.64	0.44	NA	NA	1.82	0.402	1.82	0.402	
	R^2 marginal/conditional	0.32/0.99		0.48/0.99		0.48/0.99		0.47/0.99		0.5/0.99		0.98/NA		
Total jasmonates	Veg. height	NA	NA	NA	NA	NA	NA	2.27	0.132	2.27	0.132	2.27	0.132	
	Species richness (SR)	0.83	0.361	0.83	0.361	0.82	0.366	2.32	0.128	2.32	0.128	2.02	0.155	
	Legume (L)	NA	NA	0.11	0.744	0.12	0.725	NA	NA	0.37	0.541	0.67	0.412	
	Environment (E)	1.87	0.393	1.84	0.398	1.84	0.398	1.01	0.603	0.93	0.628	0.93	0.628	
	SR x L	NA	NA	**4.45**	**0.035**	**4.45**	**0.035**	NA	NA	[3.71]	*0.054*	[3.71]	*0.054*	
	SR x E	4.00	0.136	4.03	0.134	4.31	0.116	3.37	0.186	3.45	0.178	3.65	0.161	
	L x E	NA	NA	1.75	0.416	1.47	0.479	NA	NA	1.18	0.555	0.97	0.615	
	SR x L x E	NA	NA	2.09	0.352	2.09	0.352	NA	NA	2.36	0.307	2.36	0.307	
	R^2 marginal/conditional	0.05/0.28		0.14/0.27		0.14/0.27		0.1/NA		0.19/NA		0.19/NA		
Abscisic acid (ABA)	Veg. height	NA	NA	NA	NA	NA	NA	0.58	0.445	0.58	0.445	0.58	0.445	Decreased with increasing species richness only in *P. lanceolata* phytometers in environments with
	Species richness (SR)	1.36	0.243	1.36	0.243	1.18	0.277	0.85	0.356	0.85	0.356	0.83	0.361	
	Legume (L)	NA	NA	0.03	0.852	0.22	0.641	NA	NA	0.01	0.919	0.03	0.862	
	Environment (E)	1.12	0.571	1.12	0.573	1.12	0.573	1.29	0.524	1.28	0.526	1.28	0.526	
	SR x L	NA	NA	2.20	0.138	2.20	0.138	NA	NA	2.44	0.119	2.44	0.119	

	SR x E	**5.94**	**0.048**	**5.94**	**0.048**	[4.84]	*0.089*	**6.26**	**0.044**	**6.38**	**0.041**	[5.26]	*0.072*	soil and plant history when considering the surrounding vegetation height
	L x E	NA	NA	0.38	0.828	1.52	0.467	NA	NA	0.35	0.839	1.47	0.479	
	SR x L x E	NA	NA	0.27	0.873	0.27	0.873	NA	NA	0.53	0.768	0.53	0.768	
	R^2 marginal/conditional	0.07/NA		0.1/NA		0.1/NA		0.07/NA		0.11/NA		0.11/NA		
Salicylic acid (SA)	Veg. height	NA	NA	NA	NA	NA	NA	[3.43]	*0.064*	[3.43]	*0.064*	1.00	0.317	Decreased with increasing species richness in non-legume communities. In plots with legumes, only increased with sp. richness in the environment with no history environment whereas in other environments there was no effect of species richness
	Species richness (SR)	**5.59**	**0.018**	**5.59**	**0.018**	**5.35**	**0.021**	[3.56]	*0.059*	[3.56]	*0.059*	13.41	< **0.001**	
	Legume (L)	NA	NA	0.01	0.934	0.24	0.623	NA	NA	0.23	0.629	0.14	0.707	
	Environment (E)	1.81	0.405	1.82	0.402	1.82	0.402	1.31	0.519	1.30	0.522	2.89	0.236	
	SR x L	NA	NA	**8.82**	**0.003**	**8.82**	**0.003**	NA	NA	**10.46**	**0.001**	**11.96**	**0.001**	
	SR x E	0.79	0.673	0.91	0.633	0.56	0.756	1.02	0.601	1.17	0.557	1.77	0.413	
	L x E	NA	NA	1.21	0.545	1.57	0.457	NA	NA	1.46	0.482	3.07	0.216	
	SR x L x E	NA	NA	**10.62**	**0.005**	**10.62**	**0.005**	NA	NA	**10.89**	**0.004**	**6.69**	**0.035**	
	R^2 marginal/conditional	0.16/0.33		0.29/0.35		0.29/0.35		0.18/0.33		0.31/0.34		0.02/NA		
**Iridoid glycosides**														
Aucubin	Veg. height	NA	NA	NA	NA	NA	NA	[3.56]	*0.059*	[3.56]	*0.059*	[3.56]	*0.059*	Decreased as plant species richness increased when vegetation height in the surrounding was taken into consideration
	Species richness (SR)	0.94	0.333	0.94	0.333	1.29	0.256	**6.32**	**0.012**	**6.32**	**0.012**	**5.97**	**0.015**	
	Legume (L)	NA	NA	1.98	0.160	1.63	0.202	NA	NA	0.11	0.735	0.47	0.493	
	Environment (E)	1.94	0.379	1.84	0.399	1.84	0.399	2.71	0.258	2.63	0.268	2.63	0.268	
	SR x L	NA	NA	0.04	0.840	0.04	0.840	NA	NA	0.00	0.946	0.00	0.946	
	SR x E	0.55	0.760	0.50	0.777	0.42	0.812	0.30	0.859	0.31	0.855	0.21	0.902	
	L x E	NA	NA	0.12	0.940	0.21	0.900	NA	NA	0.30	0.859	0.41	0.814	
	SR x L x E	NA	NA	3.9	0.142	3.9	0.142	NA	NA	3.75	0.153	3.75	0.153	
	R^2 marginal/conditional	0.03/0.12		0.06/0.14		0.06/0.14		0.1/NA		0.13/NA		0.13/NA		
Catalpol	Veg. height	NA	NA	NA	NA	NA	NA	**9.77**	**0.002**	**9.77**	**0.002**	**9.77**	**0.002**	Increased with increasing vegetation height
	Species richness (SR)	1.19	0.276	1.19	0.276	1.59	0.207	0.58	0.445	0.58	0.445	0.84	0.360	
	Legume (L)	NA	NA	0.43	0.510	0.03	0.866	NA	NA	**4.14**	**0.042**	**3.89**	**0.049**	
	Environment (E)	**6.27**	**0.043**	**6.26**	**0.044**	**6.26**	**0.044**	4.68	*0.097*	4.84	*0.089*	4.84	*0.089*	
	SR x L	NA	NA	0.54	0.462	0.54	0.462	NA	NA	0.60	0.440	0.60	0.440	
	SR x E	2.54	0.280	2.51	0.285	2.13	0.344	1.77	0.412	1.82	0.402	1.50	0.472	
	L x E	NA	NA	0.46	0.795	0.84	0.657	NA	NA	0.77	0.680	1.09	0.579	
	SR x L x E	NA	NA	4.07	0.131	4.07	0.131	NA	NA	2.76	0.251	2.76	0.251	
	R^2 marginal/conditional	0.07/0.12		0.1/0.16		0.1/0.16		0.12/NA		0.17/NA		0.17/NA		
**Phenylpropanoid glycosides**														
Plantamajoside	Veg. height	NA	NA	NA	NA	NA	NA	1.11	0.292	1.11	0.292	1.11	0.292	Decreased with species richness only in communities with legumes
	Species richness (SR)	0.67	0.412	0.67	0.412	0.67	0.412	0.17	0.679	0.17	0.679	0.12	0.733	
	Legume (L)	NA	NA	0.00	0.977	0.00	0.992	NA	NA	0.08	0.777	0.14	0.713	
	Environment (E)	2.99	0.225	2.99	0.224	2.99	0.224	2.63	0.268	2.58	0.276	2.58	0.276	
	SR x L	NA	NA	**9.07**	**0.003**	**9.07**	**0.003**	NA	NA	**9.07**	**0.003**	**9.07**	**0.003**	

	SR x E	3.43	0.180	3.54	0.170	2.07	0.356	3.58	0.167	3.69	0.158	2.15	0.341	
	L x E	NA	NA	1.91	0.386	3.38	0.184	NA	NA	1.89	0.388	3.43	0.180	
	SR x L x E	NA	NA	**11.04**	**0.004**	**11.04**	**0.004**	NA	NA	**10.65**	**0.005**	**10.65**	**0.005**	
	R^2 marginal/conditional	0.05/NA		0.28/NA		0.28/NA		0.04/0.26		0.28/NA		0.28/NA		
Verbascoside	Veg. height	NA	NA	NA	NA	NA	NA	0.05	0.817	0.05	0.817	0.05	0.817	Considering the presence of legumes, concentration increased with species richness in the presence of legumes but decreased in communities without legumes, regardless of the experimental environment
	Species richness (SR)	0.61	0.436	0.61	0.436	0.89	0.346	0.97	0.324	0.97	0.324	1.26	0.262	
	Legume (L)	NA	NA	0.47	0.492	0.19	0.662	NA	NA	0.28	0.595	0.00	1.000	
	Environment (E)	2.77	0.250	2.73	0.255	2.73	0.255	3.41	0.182	2.00	0.367	2.00	0.367	
	SR x L	NA	NA	**8.22**	**0.004**	**8.22**	**0.004**	NA	NA	**10.04**	**0.002**	**10.04**	**0.002**	
	SR x E	3.85	0.146	3.72	0.156	3.09	0.213	3.39	0.183	3.30	0.192	2.66	0.264	
	L x E	NA	NA	1.03	0.597	1.66	0.436	NA	NA	0.90	0.637	1.54	0.464	
	SR x L x E	NA	NA	[5.24]	*0.073*	[5.24]	*0.073*	NA	NA	**6.47**	**0.039**	**6.47**	**0.039**	
	R^2 marginal/conditional	0.06/NA		0.25/0.33		0.25/0.33		0.07/NA		0.28/NA		0.28/NA		
